# A phase I/IIa double blind single institute trial of low dose sirolimus for Pendred syndrome/DFNB4

**DOI:** 10.1097/MD.0000000000019763

**Published:** 2020-05-08

**Authors:** Masato Fujioka, Takumi Akiyama, Makoto Hosoya, Kayoko Kikuchi, Yuto Fujiki, Yasuko Saito, Keisuke Yoshihama, Hiroyuki Ozawa, Keita Tsukada, Shin-ya Nishio, Shin-ichi Usami, Tatsuo Matsunaga, Tomonobu Hasegawa, Yasunori Sato, Kaoru Ogawa

**Affiliations:** a Department of Otorhinolaryngology, Head and Neck Surgery, Keio University School of Medicine, Tokyo, Japan; b Clinical and Translational Research Center, Keio University Hospital, Tokyo, Japan; c Department of Otorhinolaryngology, Shinshu University School of Medicine, Nagano, Japan; d Department of Otolaryngology, National Hospital Organization Tokyo Medical Center, Tokyo, Japan; e Department of Pediatrics, Keio University School of Medicine, Tokyo, Japan; f Department of Preventive Medicine and Public Health, Keio University School of Medicine, Tokyo, Japan.

**Keywords:** dizziness, hearing loss, Pendred syndrome, sirolimus, therapeutics, treatment, vertigo

## Abstract

**Introduction:**

Pendred syndrome (PDS)/DFNB 4 is a disorder with fluctuating and progressive hearing loss, vertigo, and thyroid goiter. We identified pathophysiology of a neurodegenerative disorder in PDS patient derived cochlear cells that were induced via induced pluripotent stem cells and found sirolimus, an mTOR inhibitor, as an inhibitor of cell death with the minimum effective concentration less than 1/10 of the approved dose for other diseases. Given that there is no rational standard therapy for PDS, we planned a study to examine effects of low dose oral administration of sirolimus for the fluctuating and progressive hearing loss, and the balance disorder of PDS by daily monitor of their audio-vestibular symptoms.

**Methods and analysis:**

This is a phase I/IIa double blind parallel-group single institute trial in patient with PDS/DFNB4. Sixteen of outpatients with fluctuating hearing diagnosed as PDS in *SLC26A4* genetic testing aged in between 7 and 50 years old at the time of consent are given either placebo or sirolimus tablet (NPC-12T). In NPC-12T placebo arm, placebo will be given for 36 weeks; in active substance arm, placebo will be given for 12 weeks and the NPC-12T for 24 weeks. Primary endpoints are safety and tolerability. The number of occurrences and types of adverse events and of side effects will be sorted by clinical symptoms and by abnormal change of clinical test results. A 2-sided 95% confidence interval of the incidence rate by respective dosing arms will be calculated using the Clopper–Pearson method. Clinical effects on audio-vestibular tests performed daily and precise physiological test at each visit will also be examined as secondary and expiratory endpoints.

**Trial registration number:**

JMA-IIA00361; Pre-results.

## Abstract 2

Supplemental Digital Content is available in the text

**Key Points**

(1) This is the first clinical study of oral administrated drugs for genetic hearing loss.
(2) The study was designed based on preclinical proof of concept study relied on iPSCs based drug discovery and development.
(3) The study design is randomized controlled double blind parallel-group clinical trial.
(4) This study is limited to the single-site study and to patients above 7-year-old for performing audiological tests although PDS is a genetic disorder and hearing loss develops from newborn.

## 1 Introduction

Pendred syndrome (PDS)/DFNB4 is a disorder first described by Vaughan Pendred in 1896, the main symptoms of which include fluctuating and progressive hearing loss, vertigo, and goiter.^[1]^ PDS is a rare genetic disorder caused by autosomal recessive disorder, and with an estimated 4000 patients in Japan, it affects the second largest patient population among hereditary hearing disorders in the country. While the *SLC26A4* gene, which encodes PENDRIN, an anion exchanger, is known to be responsible for PDS,^[2]^ the mechanism that leads to fluctuation and progressive cochlear disorder had long remained unknown. PDS patients with fluctuating hearing loss experience volatile changes in hearing acuity, and thus they suffer not only inconveniences in their daily life, but also fear of losing their ability to communicate orally with people around them following every acute exacerbation. Symptoms of PDS involve rotating vertigo that may last a few hours to a few days once it occurs, in addition to chronic dizziness, which are grave detriments to the patients’ quality of life.^[3]^

No medication for hereditary hearing loss has pathophysiologically confirmed nonclinical POC anywhere in the world. For PDS, there is no effective treatment available at this time. The only effective interventions that exist are use of devices to augment hearing, such as hearing aids and cochlear implants. Although appropriate adjustment of a hearing aid requires consultation at a medical institution or with an audiologist, volatility in hearing and sporadic occurrence of fluctuations in a PDS patient impede the full performance of such adjustment. The hearing loss in PDS can progress as it fluctuates, and a PDS patient whose symptoms have progressed to severe hearing loss has an option to have a cochlear implant. Although the device provides a sense of sound, the sound quality is significantly deteriorated compared to what can be perceived with inner hair cells, as the number of electrodes used in a cochlear implant is limited to around twenty to date. With regard to vertigo, no medical intervention exists today, and thus patients have no option but to have bed rest and wait until the episode abates.

As described above, PDS is a rare and intractable disorder with no causal treatment, causing significant loss of patients’ QOL, and thus a new treatment has long been awaited. PDS is a hereditary disorder, and a knockout mouse for the *Slc26a4*, the responsible gene, develops severe hearing loss from severe malformation.^[4]^ A knock-in mouse for H723R mutations, the most commonly reported mutations in Japanese population with hearing impairment; however, does not develop deafness.^[5]^ This means that fluctuating and progressive hearing loss is not recapitulated in these mouse models. The lack of animal models is one of the impediments to understanding the pathology and developing treatments, and a reason that PDS treatments continue to depend on hearing devices.

We have described that this discrepancy is attributable to the species difference between primates and rodents, that is, in primates, a family protein with similar functions and SLC26A4 co-express.^[6]^ This suggests that animal models generally used to demonstrate nonclinical POC in drug discovery research are essentially inapplicable to PDS due to species difference. Considering this fact, we have undertaken preclinical studies for induced pluripotent stem cells (iPSC)-based drug discovery; identified pathophysiology of a neurodegenerative disorder that involves protein aggregation as a new pathophysiological mechanism of cochlear hearing loss; and performed drug screening with cell death suppression as metrics.^[7]^ We have discovered in this research that patient-derived iPS cells of inner ear present intracellular aggregations of mutant protein and vulnerability to cellular stress, which shares similarities in physiopathology with Parkinson disease and Alzheimer disease. We further screened existing drugs based on this finding, and identified sirolimus, an mTOR inhibitor, as an inhibitor of cell death. Oral administration of this drug has been used for 2 decades worldwide and lot of information, including adverse reaction and toxicity, has been accumulated. Common adverse effects observed among different diseases where Sirolimus was used include, for example, stomatitis, dermatitis, hypertension, and hypercholesterolemia.^[8,9]^ The minimum effective concentration required for inhibition of cell death was 0.9 ng/mL, which was less than 1/10 of the approved dose of sirolimus preparation.^[10]^

The natural history of inner ear disorder in PDS patients presents recurrent transient episodes and indolent progression. As no correlation is observed between inner ear malformation and hearing loss in PDS, hypofunction rather than malformation of the inner ear is assumed to be a therapeutic target for the disorder.

We constructed 3 different in vitro cell models that correspond to PDS pathophysiology, using the disease-specific cells generated from patient-derived iPS cells:

(1) acute injury from exposure to high-concentration epoxomicin;
(2) acute injury from exposure to low-concentration epoxomicin; and
(3) chronic injury without exposure to any agents.

Sirolimus 0.9 ng/mL significantly reduced cell death in all 3 models.^[10]^

The results suggested low-dose sirolimus for PDS patients suffering from exacerbating and progressive symptoms aggravated by life stressors and head trauma may ameliorate vulnerability of the patient's inner ear cells, and further suggested this may lead to attenuation of hearing loss and vertigo episodes and decelerated progression due to reduced cell death.

PDS is a rare disease, with an estimated patient population of around 4000 in Japan. The lack of treatment; however, motivates few patients to consult doctors regularly and willingly. Existing reports are limited to those based on limited data collected from outpatients during office visits. Use of existing historical control data on natural course of changes in hearing in PDS; therefore, is assumed to be insufficient for evaluating the efficacy in clinical research. Newly collecting and accumulating such data would thus contribute to evaluation of efficacy in this trial, and will further serve as basic information in preparing protocols for research to be conducted.

This trial will collect a larger amount of information and more detailed data by measuring objective functions of the inner ear at patients’ homes, in addition to regular office visits, and explore what tests would enable the most precise evaluation. The trial is positioned as phase I/IIa clinical research aimed at safety analysis and exploratory assessment of efficacy, including endpoints.

To explore safety, tolerability, and efficacy of NPC-12T for PDS/DFNB4, for which treatment has yet to be established.

## 2 Methods

### 2.1 Objective

To explore safety, tolerability, and efficacy of NPC-12T, 1 mg tablet of Sirolimus, for PDS/DFNB4, for which treatment has yet to be established.

### 2.2 Trial design

This is a phase I/IIa randomized, double blind parallel-group single institute trial in patient with PDS/DFNB4. The trial was designed and will be independently conducted by the Keio University with approval from the ethics committee of Keio University School of Medicine in accordance with the principals of the Declaration of Helsinki. All analyses will be conducted by Keio University, independent of the sponsor, according to the prespecified statistical analysis plan. As a prospective randomized controlled trial, the study strategy will be constructed and presented in accordance with the recommendations of the Standard Protocol Items: Recommendations for Interventional Trials statement. For the detailed protocol please refer Supplemental Digital Content:.

### 2.3 Eligibility criteria

Eligible patients are those who meet all the following inclusion criteria and who do not have any listed exclusion criteria at V0.

### 2.4 Inclusion criteria

(1) Aged at least 7 and below fifty years at the time of consent.
(2) Confirmed PDS-positive^∗^ by genetic testing such as Sanger Sequencing.
(3) Report of subjective symptoms of fluctuating hearing disorder in a medical interview for case registration.
(4) Subjective symptoms of episodes of hearing loss and/or dizziness/vertigo within 1 year before the case registration.
(5) Had undergone inner-ear function testing at least 3 times, including standard pure-tone audiometry, performed with an interval of 1 day or longer, within 6 months before the case registration.
(6) Voluntary informed consent in writing obtainable from the patient or the patient's legally acceptable representative.

^∗^Any of the pathogenic homozygous or compound-heterozygous variant can be included.

### 2.5 Exclusion criteria

(1) Prior use of molecular targeted drugs related to mTOR pathway, such as sirolimus, other mTOR inhibitors (eg, everolimus) and tyrosine kinase (eg, bevacizumab, sorafenib).
(2) History of hypersensitivity to sirolimus, including sirolimus derivatives, and its additive agents.
(3) Hepatitis B surface (HBs) antigen-positive, HBs antibody-positive, hepatitis B surface (HBc) antibody-positive, or active hepatitis C (hepatitis C virus (HCV) antibody-positive patients with inactive hepatitis and with normal-range values of liver function do not apply.) HBs antigen-positive patients for which the cause is presumed to be due to vaccination against hepatitis B may participate in this clinical trial.
(4) Severe blood abnormalities or hepatic dysfunction: aspartate transaminase (AST) or alanine transaminase (ALT) of 100 IU/L or higher, hematocrits of below 30%, Platelet count of below 80,000/mm^3^, absolute neutrophil count of below 1000/mm^3^, total white blood cell count of below 3000/mm^3^.
(5) Patients with poorly controlled dyslipidemia: serum triglyceride of 500 mg/dL or higher or low-density lipoprotein (LDL) cholesterol level of 190 mg/dL or higher despite being under treatment for dyslipidemia.
(6) Severe renal impairment: estimated glomerular filtration rate lower than 30 mL/min/1.73 m^2^.
(7) Immunodeficiency such as human immunodeficiency viruses or primary immunodeficiency.
(8) Inability to take investigational products in tablet form, or gastrointestinal dysfunction with risks of malabsorption of sirolimus
(9) History of having surgery within 8 weeks before the registration (any surgery that requires invasion of the body or suture with 3 or more stitches, including biopsy).
(10) Female patients who are pregnant, potentially pregnant, or breastfeeding. Male or female patients who decline to consent to contraception during the trial. Contraceptive methods: combination of condoms and spermicide; combination of condoms and diaphragms with spermicide; oral contraceptive; intrauterine contraceptive device; or other methods.
(11) Need to take medications that influence CYP3A4 activation after the administration of investigational products.
(12) Pulmonary interstitial opacity.
(13) Diagnosis by the attending physician during the screening phase or from previous observations from hearing tests that the hearing loss is fixed rather than fluctuating.
(14) Bilateral cochlear implant users. Note that the unilateral users are not excluded.
(15) Participation in other clinical research and/or trials within 6 months before the date of consent, with the exception of Analysis of Diseases in Otorhinolaryngology Using iPS Cells (approval No. 20140172).
(16) Other conditions that the principal investigator or subinvestigator deems inappropriate.

### 2.6 Target study sample size

A sample size of 16 subjects is expected to be studied: 12 subjects for the NPC-12T active substance arm, and 4 subjects for the NPC-12T placebo arm.

As this clinical trial will focus on a rare disease, and is exploratory, feasibility was the main aspect that was considered in setting the sample size. In terms of safety analysis, provided that clinically significant adverse events exist, the size is sufficient to detect adverse events of 15% incidence with 85% accuracy. In terms of efficacy analysis, when making a comparison of NPC-12T arms concerning hearing test endpoints of periods without treatment and with treatment, a difference can be detected at 80% power (2-sided 5% significance level), if the number of evaluable subjects is 11 and Cohen d for endpoints is 0.85. In estimation of correlation coefficient between evaluation indices, if data of 2 arms are integrated for evaluation, and the number of evaluable subjects having population correlation coefficient of 0.7 (ie, medium to high degree of correlation) is 13, then a difference can be detected at a minimum of 80% power (2-sided 5% significance level).

### 2.7 Randomization and blinding

#### 2.7.1 Methods of assignment

The randomization manager will implement random assignment in accordance with the randomization procedure. When assigning NPC-12T placebo, the randomization manager will specify 3 types of dosage: reduction, increase, or no change in dosage. In specifying reduction or increase, specific visit(s) (V7–V11) when the change in dosage is to be implemented will be indicated. The randomization manager will generate and store a randomization schedule, which is to be concealed. The randomization manager will generate prescription schedules containing information on assignment for respective subjects and specification for change in dosage of NPC-12T placebo tablets. The information for respective subjects will be sealed separately. The prescription schedules will be stored by an unblinded doctor. The randomization manager will store the randomization schedule in strict concealment until the time when the blind is permitted to be broken. When the unblinded doctor makes judgment on dosage change for a subject based on trough concentration measurements (V7–V11), the doctor will unblind the prescription schedule for the subject in question and check the criteria for dosage change. The prescription schedule will be placed in concealment again immediately after the check. The unblinded doctor will keep a record every time the prescription schedule is unblinded or concealed. The randomization manager will generate 2 sets of emergency key codes when the assignment is made, which will be kept strictly in concealment by the randomization manager and the emergency contact center (the administrative office supporting clinical trial: Keio University Hospital Clinical and Translational Research Center) until the blind is authorized to be broken.

#### 2.7.2 Indistinguishability

The randomization manager will verify the indistinguishability of medications and packages before random assignment and after the end of the trial.

#### 2.7.3 Maintenance of blindness during the trial

With regard to the blood concentration of sirolimus, any person involved in the trial, with the exception of the unblinded doctor and unblinded clinical research coordinators, will follow the procedures for prescription of investigational products to ensure blindness to the test results is maintained. The test results will be reported to the Independent Data Monitoring Committee and the principal investigator; however, after the blind is broken or if the principal investigator so directs in case of emergency. The unblinded clinical research coordinators will assist in the work of the unblinded doctor and make sure that the dosage change based on trough concentration measurements is appropriately instructed.

#### 2.7.4 Procedure for concealing and unblinding randomized code

The randomization manager will conceal and store the randomization schedule (randomized code) after the random assignment, and unblind it after the database is locked and the trial is completed.

Two sets of emergency key codes will be generated, which will be concealed by the randomization manager. The randomization manager and the emergency contact center (the administrative office supporting clinical trial: Keio University Hospital Clinical and Translational Research Center) will keep them in strict concealment until the appropriate time when the blind is permitted to be broken. When a serious adverse event or other situation demands urgent need for the principal investigator or subinvestigator to access information on assigned treatment to determine treatment of the subject, or when discovery of subject's pregnancy requires revelation of assigned treatment, the principal investigator will determine whether the code should be partially unblinded. If the principal investigator deems it necessary to unblind the code assigned to the subject in question, the emergency contact center will be requested to allow partial unblinding of the randomization code (emergency key code). The partial unblinding of the emergency key code will be conducted in accordance with the procedure manual for unblinding.

The emergency contact center will communicate the unblinded information to the principal investigator. If partial unblinding is performed, the principal investigator will document justification for unblinding and the extent to which the information is made available.

### 2.8 Registration of patients

The principal investigator or subinvestigator will obtain written informed consent from patients who are thought to satisfy the inclusion criteria, or their legally acceptable representatives, and register the patients

### 2.9 Administration of investigational products

**Figure 1:**
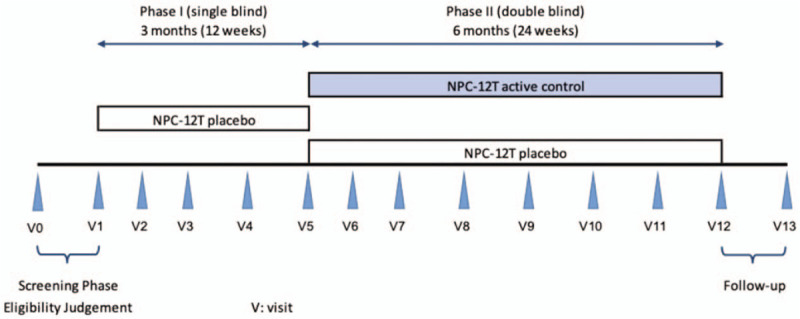
The figure details the schedule of enrolment, interventions and visits of PENDLRA trial.

The schedule for the trial visits and data collection is summarized in Figure 1. Generally, the patients will visit 14 times with screening phase for the eligibility judgement, 2 phases for the treatments, and follow-up phase.

Phase I (single-blind): NPC-12T placebo tablets for 3 months (12 weeks).

Phase II (double-blind): NPC-12T active substance tablets or NPC-12T placebo tablets for 6 months (24 weeks).

### 2.10 Tests, observations, and assessment

(1) Schedule for tests, observations, and assessment
  The items to be covered in tests and observations, and the schedule of the trial are shown in Table 1.
(2) Subjects’ baseline information
  The principal investigator or subinvestigator will collect the baseline information listed below from each subject during the period between informed consent and before administration of the investigational products in V1 (D1), and record the information on the subject's electronic case report form. For the purpose of this protocol, an illness that has not subsided before administration of the investigational products is referred to as a concomitant illness, and one that has subsided as a past medical history. The results of *SLC26A4* genetic testing will not be recorded on the electronic case report form.
(3) Concomitant medications and therapies
  The principal investigator or subinvestigator will examine concomitant medications and therapies from the screening phase (V0) to V13, and record the results on the electronic case report forms.
(4) Clinical questionnaire
  The principal investigator or subinvestigator will ask subjects to complete the questionnaire specified below (electronic Patient Reported Outcome (ePRO)) every day, in principle, from the screening phase (V0) to V13, and record the results on the electronic case report forms.
  Questionnaire on daily conditions: medication adherence, hearing loss episodes, vertigo episodes, tinnitus, ear fullness, concomitant medications, handicaps caused by dizziness/vertigo (action, feeling, and daily life)
  The principal investigator or subinvestigator will ask subjects to complete the Dizziness Handicap Inventory at every visit from the screening phase (V0) to V13 and at discontinuation, and record the results on the electronic case report forms.
(5) Tests using portable testing devices
  The principal investigator or subinvestigator will provide subjects training on the portable devices and make adjustments to the devices during the screening phase (V0). Subjects will be asked to perform tests using the portable devices listed below from V0 to V13 every night, in principle. The test results will be collected at every visit from V1 to V13 and at discontinuation.

**Table 1:**
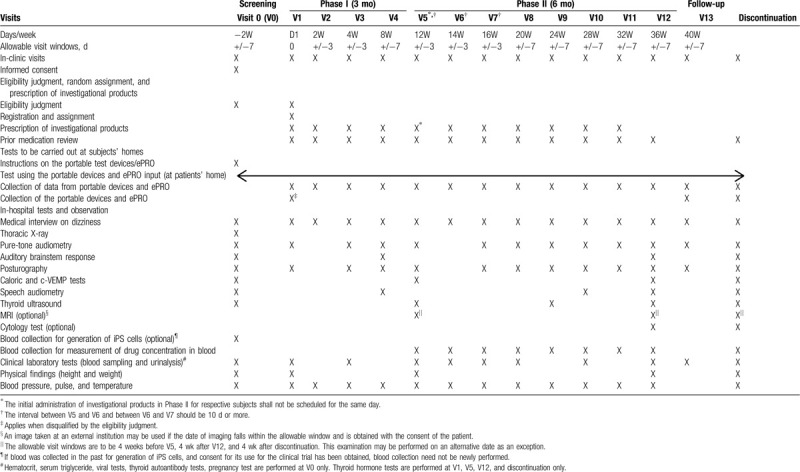
Procedures and schedule.

Portable devices used are below:

1. Portable audiometer: Audiometer AA-58 (RION Co., Ltd.: 3-20-41 Higashimotomachi, Kokubunji, Tokyo, 185-8533, Japan.)
  For standard pure-tone audiometry (threshold of 4 different frequencies for left and right ears) and recording data in ePRO. The frequencies include 500, 1000, 2000, and 4000 Hz.
2. Wireless Frenzel scope: AirMicro Frenzel Wireless Scope (Scalar Corporation: San-Ei Building 1F, Nishi-Shinjuku 1-22-2, Shinjuku-ku,Tokyo 160-0023 Japan)
  For tests such as measurement of rotational eye movement, and direction and amplitude of nystagmus, the results of which shall be transmitted to an Android terminal for storage. The tests will be performed regularly in the evening and as appropriate when an episode occurs, as far as possible.
3. Head stability test: JINS MEME ES_R (JIN Co.,Ltd.: Iidabashi Grand Bloom 30F, 2-10-2, Fujimi, Chiyoda-ku, Tokyo 102-0071, Japan.)
  For tests with open and closed eyes, each lasting around 30 seconds, the results of which shall be transmitted to an Android terminal for storage.
  (6) In-clinic testing and observations
  Vital signs, physical findings, general laboratory tests (Table 2), thoracic radiography, pure-tone audiometry, auditory brainstem response, posturography, caloric test, cervical vestibular evoked myogenic potential (c-VEMP) test, speech audiometry, and blood concentration of Sirolimus will be tested in all participants.
  (7)Exploratory tests and observations
  1. Collection of blood for generation of iPS cells and in vitro efficacy testing of sirolimus (optional)
    The principal investigator or subinvestigator will collect blood to be used for generation of iPS cells. Generation of iPS cells and in vitro efficacy testing using PDS-specific iPS cells will be performed at the Keio University School of Medicine.
  2. Thyroid ultrasound
  3. Magnetic resonance imaging (MRI) (optional)
    MRI scan of the inner ear will be performed during phase II and at discontinuation for subjects aged 16 or older with consent to the test, 4 hours after intravenous injection of contrast media.^[11]^
  4. Thyroid cytology test (optional)
    Cytology tests of thyroid for an exhaustive gene expression analysis in phase II (V5 and V12) and at discontinuation for subjects with consent
  (6) Adverse events
  1. Definition of adverse events
    An adverse event in this trial refers to any untoward medical occurrence in a subject from initial administration of investigational products (V1) to follow-up observations (V13). The event may not necessarily have to have a causal relationship with administration of investigational products in this trial.
  2. Definition of serious adverse events
    Seriousness of adverse events is categorized as follows:
    1) Nonserious: cases other than 2)
    2) Serious: events for which any of the items from (a) to (g), below, apply. (a) Death, (b) Cases that might result in death, (c) Any case that requires hospitalization for treatment or prolongs the duration of hospitalization, (d) Disability, (e) Cases that might result in disability, (f) Other medically serious condition for which seriousness is equivalent to (a) to (e), above (g) Any congenital diseases or abnormalities in the next generation

**Table 2:**
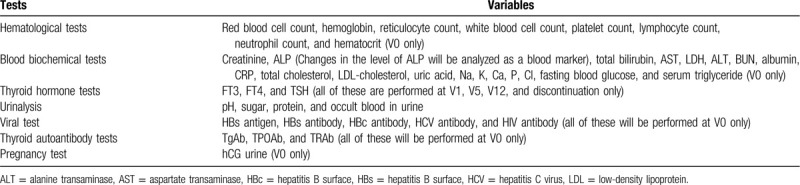
Laboratory test items.

### 2.11 Efficacy endpoints

Assessment of efficacy in this trial is regarded as a secondary objective. Following will be examined.

#### 2.11.1 Secondary endpoints

(1) Frequency of hearing loss episodes (number of occurrences per year).
(2) Highest measurement of auditory threshold shift at a hearing loss episode (dB) Definition of hearing loss episodes: Hearing loss episodes with subjective symptoms and episodes detected as audiometric data will be counted as hearing loss episodes in this trial. Concerning audiometric data, audiometric testing of 4 different frequencies for right and left ears will be performed, and the average of measurements obtained at 5 recent time points will be defined as the baseline. A change of 10 dB or more observed in the testing period including the screening phase will be identified as an episode. The period from the beginning of the episode until the condition returns to the baseline will be counted as 1 episode. Episodes observed in left and right ears or in different frequencies concurrently will be considered as separate episodes.
(3) Frequency of dizzy spells (number of occurrences per year) Dizzy spells in which the subject is conscious are to be counted.
(4) Maximum amplitude (°/s) and maximum frequency (cycle/s) of nystagmus in a hearing loss episode
(5) Percentage of cases in which increase in hearing threshold was observed during remission in the observation period (comparison of measurements at the end of phase I and the end of phase II).
(6) Improvement in threshold during remission.
(7) Shorter duration of hearing loss episodes.
(8) Alleviation of symptoms of dizziness/vertigo Changes in total scores of the Dizziness Handicap Inventory from phase I to phase II.
(9) Percentage of cases in which equilibrium exacerbated during remission in the observation period (comparison of measurements at the end of Phase I and the end of Phase II).

#### 2.11.2 Exploratory endpoints

1. Reduction in endolymphatic hydrops (MRI findings).
2. Inhibition of goiter or thyroid enlargement.
3. Cell biological changes in thyrocytes detected by cytology tests.
4. Comparison of results of in vitro efficacy evaluation of inner ear cells derived from PDS-specific iPS cells and of clinical evaluation.
5. Deviation from threshold value for frequency of abnormalities of auditory brain stem responses and for pure-tone audiometry.

### 2.12 Statistics

Primary endpoints are safety and tolerability. The number of occurrences and types of adverse events and of side effects will be sorted by clinical symptoms and by abnormal change of clinical test results. A 2-sided 95% confidence interval of the incidence rate by respective dosing arms will be calculated using the Clopper–Pearson method.

Assessment of efficacy in this trial is regarded as a secondary objective. With regard to continuous endpoints, summary statistics of changes or percentage changes based on actual measurements and from baseline at each point in time will be calculated for each dosage arm. A 1-sample *t* test will be used for comparison between actual measurements and baseline values at each point in time for each dosage arm. The 2-sided 95% confidence interval for average change for each dosage arm will then be estimated. Using a 2-sample *t* test, the change or percent change at each point in time between the 2 arms will be compared. The 2-sided 95% confidence interval for average difference between the 2 arms will be estimated. If the data is found to be non-normal, use of nonparametric methods and statistical methods based on probability distribution other than normal distribution will be considered. With regard to binary endpoints, a contingency table will be compiled for each point in time, and Fisher exact test will be applied in making comparison of the distribution in the 2 dosage arms. The Clopper–Pearson method will be applied to estimate the 2-sided 95% confidence interval for each dosage arm. The difference in the 2-sided 95% confidence interval will be estimated using the normal approximation.

### 2.13 Patient and public involvement

Patients or the public were not directly involved in the research. Nevertheless, the main results will be made available in the public domain.

### 2.14 Ethics and dissemination

Participant recruitment began in May 2018. The final results will be published in international peer-reviewed medical journals.

## 3 Discussion

PDS is a disorder with fluctuating and progressive hearing loss, vertigo, and thyroid goiter and there is so far no rational treatment. We identified pathophysiology of a neurodegenerative disorder that involves protein aggregation in PDS patient derived cochlear cells that were induced via iPSCs and found an mTOR inhibitor sirolimus as a potential therapeutic target. Also, we identified minimum effective concentration less as an inhibitor of cell death by using iPSCs derived disease cells, with the than 1/10 of the approved dose for other diseases. So far as we searched, this is the first study starting clinical trial with the initial Phase I/II trial defined by iPSCs based preclinical proof of concept study. The result will strengthen the concept of iPSCs based drug development itself if we obtained good outcomes. The optional expiratory trial examining correlation in between in vitro effect and actual patient's’ clinical effect may also support the idea.

Daily monitoring for audio-vestibular symptoms will be carried out. The result will offer natural time-course of fluctuation in patient with PDS, which has never been reported. Also, the system itself can be applied for other hearing and balance disorders. In addition, episodes in between the patients’ visits, including traumas, are asked carefully in every visit by the coordinators and investigators. In each visit, first the investigators briefly review the data-history collected at home in between visits. Then they ask patients’ daily life-events, especially those at the specific days when the hearing loss and vertigo was documented. We believe the Internet of Things (IoT)-based data collection system may facilitate medical staffs understand the clinical features of individuals more efficiently and accurately.

Limitation of the study is that we will only examine patients above 7-year-old because performing audiological tests would be difficult for the younger ages. PDS is a genetic disorder and hearing loss develops from newborn. Further clinical trials will have to be awaited in the future to know the early stage development of PDS.

## Acknowledgments

The authors thank Takayuki Abe, former staff member of Department of Preventive Medicine and Public Health, Keio University School of Medicine, Tokyo, Japan for statistical analyses and Yasuko Ochi a member of the Department of Otorhinolaryngology in Keio University School of Medicine for their help in the preparation of this manuscript.

## Author contributions

Masato Fujioka wrote the manuscript. Masato Fujioka, Takumi Akiyama, Makoto Hosoya, Kayoko Kikuchi, Yuto Fujiki, Yasuko Saito, Keita Tsukada, Hiroyuki Ozawa, Tomonobu Hasegawa,

Kaoru Ogawa planned the trial protocol. Masato Fujioka, Keisuke Yoshihama, Shin-Ya Nishio, Shin-Ichi Usami, Tatsuo Matsunaga, Yasunori Sato evaluated basic information regarding genetic tests and calculate affordable sample size. YS planned statistical analyses.

## Supplementary Material

Supplemental Digital Content
