# Supplementary material for: A phase I/IIa double blind single institute trial of low dose sirolimus for Pendred syndrome/DFNB4
Source: Medicine (Baltimore). 2020 May 8;99(19):e19763. doi: 10.1097/MD.0000000000019763 (PMC7220392; doi:10.1097/MD.0000000000019763)
Supplement: Supplemental Digital Content [file medi-99-e19763-s001.docx]

CLINICAL TRIAL PROTOCOL

A Phase I/IIa double blind single institute trial of low dose sirolimus for Pendred syndrome/ DFNB4

Sponsor-Investigator (Principal Investigator): Masato Fujioka

Department of Otorhinolaryngology, Head and Neck Surgery, Keio University School of Medicine

This document is confidential and remains the sole property of the sponsor-investigator. No unpublished information contained in this document may be disclosed without the prior written consent of the sponsor-investigator.

Protocol Number: KCTR-D012

Version Number: v. 11.0.0

May 16, 2019

Table of Contents

[1. List of Abbreviations 6](#_Toc20404146)

[2. Protocol Summary 8](#_Toc20404147)

[3. Background and Rationale 20](#_Toc20404148)

[3.1. Pendred syndrome/DFNB4 20](#_Toc20404149)

[3.2. Challenges in PDS Research and Background of Development of This Therapy (iPS Drug Discovery) 20](#_Toc20404150)

[3.3. Problems in Clinical Development for PDS Inner Ear Disorder 21](#_Toc20404151)

[4. Objective 22](#_Toc20404152)

[5. Study Plan 22](#_Toc20404153)

[5.1. Schedule of Activities 22](#_Toc20404154)

[5.1.1. Expected Duration of the Study 25](#_Toc20404155)

[5.1.2. Period for Administering the Investigational Products 25](#_Toc20404156)

[5.1.3. Clinical Trial Design 25](#_Toc20404157)

[5.2. Study Population 25](#_Toc20404158)

[6. Endpoints 27](#_Toc20404159)

[7. Target Study Sample Size 28](#_Toc20404160)

[8. Informed Consent Process 28](#_Toc20404161)

[8.1. Preparation of Written Information and Consent Form 28](#_Toc20404162)

[8.2. Elements to be Included in the Informed Consent Document 28](#_Toc20404163)

[8.3. Procedure for Obtaining Informed Consent 29](#_Toc20404164)

[8.4. Revision of the Informed Consent Form 30](#_Toc20404165)

[9. Investigational Products 30](#_Toc20404166)

[9.1. Brief Description of the Investigational Products 30](#_Toc20404167)

[9.2. Packaging and Labeling 31](#_Toc20404168)

[9.3. Handling of the Investigational Products 33](#_Toc20404169)

[10. Procedures of the Trial 33](#_Toc20404170)

[10.1. Registration of Patients 33](#_Toc20404171)

[10.2. Randomization and Blinding 34](#_Toc20404172)

[10.3. Procedure for Concealing and Unblinding Randomized Code 35](#_Toc20404173)

[11. Administration of Investigational Products 36](#_Toc20404174)

[12. Prohibited Concomitant Medications and Therapies 39](#_Toc20404175)

[13. Oversight of Subjects 40](#_Toc20404176)

[14. Tests, Observations, and Assessment 40](#_Toc20404177)

[14.1. Schedule for Tests, Observations, and Assessment 40](#_Toc20404178)

[14.2. Subjects’ Baseline Information 41](#_Toc20404179)

[14.3. Concomitant Medications and Therapies 41](#_Toc20404180)

[14.4. Medication Adherence 41](#_Toc20404181)

[14.5. Clinical Questionnaire 41](#_Toc20404182)

[14.6. Tests Using Portable Testing Devices 42](#_Toc20404183)

[14.7. In-clinic Testing and Observations 42](#_Toc20404184)

[14.7.1. Vital Signs 42](#_Toc20404185)

[14.7.2. Physical Findings 43](#_Toc20404186)

[14.7.3. Laboratory Tests 43](#_Toc20404187)

[14.7.4. Thoracic Radiography 43](#_Toc20404188)

[14.7.5. Pure-tone Audiometry 43](#_Toc20404189)

[14.7.6. Auditory Brainstem Response 43](#_Toc20404190)

[14.7.7. Posturography 44](#_Toc20404191)

[14.7.8. Caloric Test 44](#_Toc20404192)

[14.7.9. c-VEMP Test 44](#_Toc20404193)

[14.7.10. Speech Audiometry 44](#_Toc20404194)

[14.8. Blood Concentration of Sirolimus 44](#_Toc20404195)

[14.9. Exploratory Tests and Observations 44](#_Toc20404196)

[14.9.1. Collection of Blood for Generation of iPS Cells and in vitro Efficacy Testing of Sirolimus (Optional) 44](#_Toc20404197)

[14.9.2. Thyroid Ultrasound 45](#_Toc20404198)

[14.9.3. MRI (optional) 45](#_Toc20404199)

[14.9.4. Cytology Test (optional) 45](#_Toc20404200)

[14.10. Adverse Events 45](#_Toc20404201)

[14.10.1. Definition of Adverse Events 45](#_Toc20404202)

[14.10.2. Assessment of Adverse Events 45](#_Toc20404203)

[14.10.3. Definition of Serious Adverse Events 46](#_Toc20404204)

[14.10.4. Causality Assessment 46](#_Toc20404205)

[14.10.5. Severity of Adverse Event 46](#_Toc20404206)

[14.10.6. Reporting of Serious Adverse Events 47](#_Toc20404207)

[14.10.7. Follow-up of Adverse Events 47](#_Toc20404208)

[14.10.8. Provision of Additional Information 47](#_Toc20404209)

[14.10.9. Pregnancy during the Trial 48](#_Toc20404210)

[14.11. Discontinuation Criteria 48](#_Toc20404211)

[14.11.1. Participant Withdrawal 48](#_Toc20404212)

[14.11.2. Discontinuation of the Clinical Trial 49](#_Toc20404213)

[15. Independent Data Monitoring Committee 49](#_Toc20404214)

[15.1. Establishment of the Independent Data Monitoring Committee 49](#_Toc20404215)

[15.2. Purpose of the Independent Data Monitoring Committee 49](#_Toc20404216)

[15.3. Assembly of the Independent Data Monitoring Committee 49](#_Toc20404217)

[15.4. Meeting Format and Discussion Materials for the Independent Data Monitoring Committee 50](#_Toc20404218)

[15.5. Criteria of Judgment by the Independent Data Monitoring Committee 50](#_Toc20404219)

[16. Compliance with, Deviation from, Changes in, or Amendment to the Protocol 51](#_Toc20404220)

[16.1. Compliance with the Protocol 51](#_Toc20404221)

[16.2. Protocol Deviations or Changes 51](#_Toc20404222)

[17. Statistical Analyses 51](#_Toc20404223)

[17.1. Populations for Analyses 51](#_Toc20404224)

[17.1.1. Analysis Sets 51](#_Toc20404225)

[17.1.2. Data Handling 52](#_Toc20404226)

[17.2. Demographic Variables and Baseline 52](#_Toc20404227)

[17.3. Statistical Analysis of Efficacy 52](#_Toc20404228)

[17.3.1. Efficacy Endpoints 53](#_Toc20404229)

[17.4. Statistical Analysis of Safety 54](#_Toc20404230)

[17.4.1. Adverse Events 54](#_Toc20404231)

[17.4.2. Clinical Test Results and Vital Signs 54](#_Toc20404232)

[17.5. Target Study Sample Size 54](#_Toc20404233)

[17.6. Significance Level and Multiplicity 54](#_Toc20404234)

[17.7. Interim Analyses 54](#_Toc20404235)

[17.8. Deviations from the Original Statistical Analysis Plan 55](#_Toc20404236)

[17.9. Statistical Analysis Plan 55](#_Toc20404237)

[18. Electronic Case Report Forms 55](#_Toc20404238)

[18.1. Entry into and Reporting via the Electronic Case Report Forms 55](#_Toc20404239)

[18.2. Principal Investigator’s Confirmation of the Electronic Case Report From 55](#_Toc20404240)

[18.3. Database Locking and Unlocking 55](#_Toc20404241)

[18.4. Entry in the Questionnaire and Testing and Report Using a Portable Device 56](#_Toc20404242)

[18.5. Materials Recorded Directly on the Case Report Forms and Materials Regarded as Source Data/Documents 56](#_Toc20404243)

[18.6. Direct Access to Source Data/Documents 56](#_Toc20404244)

[19. Quality Management and Assurance 56](#_Toc20404245)

[20. Ethical Considerations 57](#_Toc20404246)

[20.1. Institutional Review Board 57](#_Toc20404247)

[20.2. Patient Confidentiality 57](#_Toc20404248)

[21. Record Keeping 57](#_Toc20404249)

[22. Financial assistance 58](#_Toc20404250)

[23. Compensation for Adverse Health Effects 58](#_Toc20404251)

[24. Publication Policy 59](#_Toc20404252)

[25. References 59](#_Toc20404253)

[26. Organization of the Trial Site 59](#_Toc20404254)

[27. List of Annex 59](#_Toc20404255)

# List of Abbreviations

| Abbreviations | Full form |
| --- | --- |
| ADL | activity of daily living |
| ALP | alkaline phosphatase |
| ALT | alanine transaminase |
| AMED | Japan Agency for Medical Research and Development |
| AST | aspartate transaminase |
| ATP | adenosine triphosphate |
| AUC | area under the plasma concentration-time curve |
| BUN | blood urea nitrogen |
| Cmax | maximum concentration |
| CRO | contract research organization |
| CRP | C-reactive protein |
| cVEMP | cervical vestibular evoked myogenic potential |
| CYP | cytochrome P-450 |
| DHI | Dizziness Handicap Index |
| eGFR | estimate glomerular filtration rate |
| ePRO | electronic Patient Reported Outcome |
| FAS | full analysis set |
| FT3 | free triiodothyronine |
| FT4 | free thyroxine |
| GCP | Good Clinical Practice |
| HBs | hepatitis B surface |
| HCV | hepatitis C virus |
| HIV | human immunodeficiency virus |
| iPS | induced pluripotent stem |
| ITT | intention-to-treat |
| LDH | lactate dehydrogenase |
| LDL | low-density lipoprotein |
| MRI, MR | magnetic resonance imaging |
| mTOR | mammalian target of rapamycin |
| PDS | Pendred syndrome/DFNB4 |
| POC | proof of concept |
| PPS | per protocol set |
| QOL | quality of life |
| SDV | source document verification |
| TgAb | anti-thyroglobulin antibody |
| TPOAb | anti-thyroid peroxidase antibody |
| TRAb | TSH receptor antibody |
| TSH | thyroid-stimulation hormone |

# Protocol Summary

| Synopsis |
| --- |
| 1. Investigational Products   Test drug: NPC-12T active substance tablets  Comparator: NPC-12T placebo tablets |
| 1. Title   A Phase I/IIa double blind parallel-group single institute trial of low dose sirolimus for Pendred syndrome/ DFNB4 |
| 1. Objective   To explore safety, tolerability, and efficacy of NPC-12T for Pendred syndrome/DFNB4 (PDS), for which treatment has yet to be established |
| 1. Endpoints   [Primary Endpoints]  Safety and tolerability:  1) Adverse events (nature, frequency, and severity)  2) Side effects (nature, frequency, and severity)  3) Clinical laboratory tests  4) Vital signs (blood pressure, pulse, and temperature)  [Secondary Endpoints]  Efficacy:  1) Frequency of hearing loss episodes (number of occurrences/year)  2) Highest measurement of auditory threshold shift at a hearing loss episode (dB)  3) Frequency of dizzy spells (number of occurrences/year)  4) Maximum amplitude (°/sec) and maximum frequency (cycle/sec) of nystagmus during a hearing loss episode  5) Percentage of cases in which increase in hearing threshold was observed during remission in the observation period from the end of Phase I to the end of Phase II  6) Improvement in hearing threshold during remission  7) Shorter duration of hearing loss episodes  8) Alleviation of symptoms of dizziness/vertigo  Changes in total scores of the Dizziness Handicap Inventory from Phase I to Phase II  9) Percentage of cases in which equilibrium exacerbated during remission in the observation period from the end of Phase I to the end of Phase II  [Exploratory Endpoints]  1) Reduction in endolymphatic hydrops (MRI findings)  2) Inhibition of goiter or thyroid enlargement  3) Cell biological changes in thyrocytes detected by cytology tests  4) Comparison of results of in vitro efficacy evaluation of inner ear cells derived from PDS-specific iPS cells and of clinical evaluation  5) Deviation from threshold value for frequency of abnormalities of auditory brain stem responses and for pure-tone audiometry |
| 1. Study Design   A phase I/IIa double-blind, parallel-group, single-institute trial initiated by a medical investigator |
| 1. Inclusion and Exclusion Criteria   Outpatients with fluctuating hearing diagnosed as PDS in *SLC26A4* genetic testing are eligible to participate.  [Inclusion Criteria]  1) Aged at least seven and below fifty years at the time of consent  2) Confirmed PDS-positive by genetic testing such as Sanger Sequencing  3) Report of subjective symptoms of fluctuating hearing disorder in a medical interview for case registration  4) Subjective symptoms of episodes of hearing loss and/or dizziness/vertigo within one year prior to the case registration  5) Had undergone inner-ear function testing at least three times, including standard pure-tone audiometry, performed with an interval of one day or longer, within six months prior to the case registration  6) Voluntary informed consent in writing obtainable from the patient or the patient’s legally acceptable representative  [Exclusion Criteria]  1) Prior use of molecular targeted drugs related to mTOR pathway, such as sirolimus, other mTOR inhibitors (e.g., everolimus) and tyrosine kinase (e.g., bevacizumab, sorafenib)  2) History of hypersensitivity to sirolimus, including sirolimus derivatives, and its additive agents  3) HBs antigen-positive, HBs antibody-positive, HBc antibody-positive, or active hepatitis C (HCV antibody-positive patients with inactive hepatitis and with normal-range values of liver function do not apply.)  HBs antigen-positive patients for which the cause is presumed to be due to vaccination against hepatitis B may participate in this clinical trial.  4) Severe blood abnormalities or hepatic dysfunction that demonstrate conditions shown below   \| Items \| Value \| \| --- \| --- \| \| AST or ALT \| 100 IU/L or higher \| \| Hematocrits \| below 30% \| \| Platelet count \| below 80,000/mm^3^ \| \| Absolute neutrophil count \| below 1,000/mm^3^ \| \| Total white blood cell count \| below 3,000/mm^3^ \|   5) Patients with poorly controlled dyslipidemia  Serum triglyceride of 500 mg/dL or higher or LDL cholesterol level of 190 mg/dL or higher despite being under treatment for dyslipidemia  6) Severe renal impairment  Estimated glomerular filtration rate (eGFR) lower than 30 mL/min/1.73 m^2^  7) Immunodeficiency such as HIV or primary immunodeficiency  8) Inability to take investigational product in tablet form, or gastrointestinal dysfunction with risks of malabsorption of sirolimus  9) History of having surgery within eight weeks prior to the registration (any surgery that requires invasion of the body or suture with three or more stitches, including biopsy)  10) Female patients who are pregnant, potentially pregnant, or breastfeeding. Male or female patients who decline to consent to contraception during the trial  11) Need to take medications that influence CYP3A4 activation after the administration of investigational product  12) Pulmonary interstitial opacity  13) Diagnosis by the attending physician during the screening phase or from previous observations from hearing tests that the hearing loss is fixed rather than fluctuating  14) Participation in other clinical research and/or trials within six months prior to the date of consent, with the exception of Analysis of Diseases in Otorhinolaryngology Using iPS Cells (approval No. 20140172)  15) Other conditions that the principal investigator or subinvestigator deems inappropriate |
| 1. Dosage of the Investigational Products     Dosing Protocol  Phase I (single-blind): NPC-12T placebo tablets for 3 months (12 weeks)  Phase II (double-blind): NPC-12T active substance tablets or NPC-12T placebo tablets for 6 months (24 weeks)  [Dosage at the outset of the trial]  Subjects weighing less than 40 kg will take one 1-mg tablet orally once every other day.  Subjects weighing 40 kg or more will take one 1-mg tablet orally once per day.  The investigational products are to be taken before sleep.  The bodyweight measured at V1 will be referred to in determining dosage throughout the trial.  [Prescription of the investigational products]  In Phase I (V1, V2, V3, and V4) and Phase II (V5 and V6), the principal investigator or subinvestigator will prescribe investigational products in accordance with the dosage at the start of the trial described above.  In Phase II (V7, V8, V9, V10 and V11), an unblinded doctor will instruct the principal investigator and subinvestigators on the prescription of the investigational product based on the criteria for dosage change. The principal investigator or subinvestigator will make a judgment on whether dosage reduction is required or dosage increase is permissible, taking account of subjects’ safety, including risks of adverse events. If the conditions are deemed appropriate, the principal investigator or subinvestigator will prescribe the investigational products as instructed by the unblinded doctor. The unblinded doctor will provide instructions to the principal investigator or subinvestigator in accordance with the trough concentration measurements and the prescription schedule generated by the randomization manager. The trough concentration measurements and the prescription schedule are not to be disclosed to any person except the unblinded CRCs until the end of the trial, and will be kept securely locked in a cabinet accessible only to the unblinded doctor.  [Criteria for dosage change]  From Phase II, the dosage will be determined taking account of the trough concentration measurements from the last visit (V6, V7, V8, V9, and V10) and occurrence of adverse events.  The unblinded doctor will provide instructions to the principal investigator or subinvestigator on prescription of the investigational products based on the trough concentration measurements at visits.  The principal investigator or subinvestigator will make a judgment on whether dosage reduction is required or dosage increase is permissible before prescribing, with due consideration for the subjects’ safety, such as risks of adverse events.  In this trial, the therapeutic range of the trough concentration following the administration of NPC-12T active substance is set to be 2.5 to 7.5 ng/mL. The trough concentration at the last visit below 2.5 ng/mL will be considered to be suitable for dosage increase, and that exceeding 7.5 ng/mL for dosage reduction.  Dosage increase for NPC-12T active substance will be permitted only if the principal investigator or subinvestigator deems it appropriate from the viewpoint of subject safety. The unblinded doctor will instruct the principal investigator or subinvestigator to prescribe the investigational products not exceeding two 1-mg tablets per day (i.e., 2 mg/day), administered orally.  The reduced dosage of NPC-12T active substance should be no less than one 1-mg tablet every three days, administered orally. If further reduction is required, administration of the investigational product to the subject will be discontinued.  As adjustment of dosage according to trough concentration is unsuitable for the NPC-12T placebo arm, one of increase, reduction, or no change in dosage is specified for the placebo arm at the time of assignment of the investigational products. At the same time, the randomization manager will designate the visits for dosage change for the placebo arm.  Dosage increase for NPC-12T placebo will be permitted only if the principal investigator or subinvestigator deems it appropriate from the viewpoint of subject safety. When dosage increase is specified in the prescription schedule, the unblinded doctor will instruct the principal investigator or subinvestigator to prescribe a dosage level at one tier higher.  If the prescription schedule specifies reduction, the unblinded doctor will instruct the principal investigator or subinvestigator regarding dosage reduction for NPC-12T placebo. The dosage level will be lowered by one tier at a time. The reduced dosage must be no lower than one 1-mg tablet every three days, administered orally. If the principal investigator or subinvestigator determines further reduction is required, administration of the investigational products will be discontinued.  The unblinded doctor will provide instructions to the principal investigator or subinvestigator on prescription in accordance with the specified dosage change in the prescription schedule. The decision by the principal investigator or subinvestigator on permission for dosage increase and reduction shall take precedence over the specification in the prescription schedule, i.e., if the principal investigator or subinvestigator specifies reduction, and the prescription schedule states increase, reduction should be adopted.  [Acceptable change in dosage]  The amounts of investigational products to be administered can be increased or decreased to one of the following dosages:  A. Two 1-mg tablets, once per day, administered orally  B. One 1-mg tablet, once per day, administered orally  C. One 1-mg tablet, once every other day, administered orally  D. One 1-mg tablet, once every three days, administered orally  [Dosage reduction or increase]  When dosage is to be increased, the dosage level should be raised by one tier at a time: Subjects weighing 40 kg or over will follow the change from B to A. Subjects weighing below 40 kg will follow the change from C to B to A.  When dosage is to be reduced, the dosage level should be lowered by one tier at a time, from A to B to C to D. If the current level for a subject is B, for example, C will be applied in the next reduction. If further reduction below D is required, administration of the investigational product to the subject will be discontinued.  [Suspension or reduction of administration of investigational products due to adverse events]  ・Unacceptable adverse events or exacerbation considering the safety of the subject  ・Unacceptable abnormal observations in clinical tests considering the safety of the subject  If a situation described above arises, dosing will be suspended until the relevant symptoms abate. The dosage will then be reduced by one tier at a time from A to B to C to D. If the sirolimus maintenance dose is 1 mg per day every three days, administered orally, which corresponds to D, the dosing will be discontinued, and follow-up observations will be performed one month after that, before discontinuation of the patient’s participation in the trial, in principle.  The initial administration of investigational products in Phase II for respective subjects shall not be performed on the same day. |
| 1. Prohibited Concomitant Medications and Therapies   1) In principle, subjects will be prohibited from taking any of the following medications unless the principal investigator or subinvestigator allows the concomitant use in response to acute exacerbation:  ATP solution (e.g., Adetphos^Ⓡ^), Vitamin B12 solution (e.g., Methycobal^Ⓡ^), Betahistine (e.g., Merislon), and medications that improve microcirculation (e.g., Kallikrein).  2) Subjects will be prohibited from concomitant use of medications described below.  Sirolimus is metabolized by a drug-metabolizing enzyme, CYP3A4, and is a substrate for P-glycoprotein. Sirolimus itself is an inhibitor of CYP3A4. Subjects are thus prohibited from taking any of the systemic medications and foods listed below throughout their participation in this clinical trial, i.e., from the initial administration of investigational products (V1) to the end of the administration (V12).   \| Influencing factors \| Medications and foods \| Rationale \| \| --- \| --- \| --- \| \| 1) Inhibition of mTOR \| everolimus; temsirolimus \| The products have the same mechanism of action as the investigational products, which would disturb efficacy analysis. \| \| 2) Influence on the assessment of the disease studied \| aminoglycoside class of antibiotics; platinum-based drugs \| Ototoxicity of the products could exacerbate inner ear disorder. (Topical administration is allowed.) \| \| 3) Live vaccines \| live vaccines (e.g., measles, mumps, rubella, oral polio, BCG) \| The immunosuppresive properties of sirolimus may cause proliferation of microorganisms in live vaccines leading to enhanced virulence.  (The package insert of Rapamune states these as contraindications for concomitant use.) \| \| 4) Induction of CYP3A4 \| anticonvulsants (e.g., carbamazepine, phenobarbital, phenytoin); rifampicin*; rifabutin; foods containing St. John’s Wort (*Hypericum perforatum*) \| The products induce CYP3A4 and may lower blood concentrations of the investigational products.  (*Rifampicin induces P-glycoprotein and thus may stimulate removal of the investigational products from cells.) \| \| 5) Inhibition of CYP3A4 \| ciclosporin**; diltiazem**, triazole antifungal (e.g., fluconazole, ketoconazole**, itraconazole, voriconazole); HIV protease inhibitors (e.g., indinavir, ritonavir, telaprevir, saquinavir); macrolide class of antibiotics (e.g., erythromycin, clarithromycin); calcium channel blocker (e.g., nicardipine, verapamil); cimetidine; metoclopramide; bromocriptine; danazol; grapefruit juice \| The products significantly inhibit CYP3A4, and may raise blood concentrations of the investigational products. (Topical administration is allowed.)  (**These products inhibit P-glycoprotein and thus may hinder removal of the investigational products from cells.) \| \| 6) Inhibition of CYP3A4 by ritonavir \| ombitasvir hydrate; paritaprevir hydrate; ritonavir \| Inhibition of CYP3A4 by ritonavir may raise AUC and C_max_ of the investigational products. \| \| 7) Mechanism unidentified \| micafungin sodium \| The product may increase AUC of the investigational products. \| \| 8) Other effects \| angiotensin-converting-enzyme inhibitor \| For a patient taking medications related to angioedema, the product may increase risks of onset of angioedema (e.g., swelling of face, lips, tongue, or throat). (Mechanism unidentified) \| |
| 1. Discontinuation of the Clinical Trial or Subject Participation in the Trial   [Participant Withdrawal]  Subjects who meet any of the criteria listed below will be withdrawn from the trial.  1) Subject requests withdrawal from the trial  2) Subject no longer trackable  3) Discovery of obvious ineligibility for participation in the trial  4) Decision of the principal investigator or subinvestigator that continuation of the subject’s participation in the trial would be difficult due to an adverse event that occurs after the initiation of the trial  5) Pregnancy of the subject  6) Death of the subject  7) Need for dosage reduction below the lowest standard of one 1-mg tablet every three days, administered orally  8) Other conditions that the principal investigator or subinvestigator deems appropriate for withdrawal  [Discontinuation of the Clinical Trial]  1) The Independent Data Monitoring Committee will be convened to review appropriateness of continuation of the trial, if adverse events of the following types occur: one or more serious adverse events, regardless of causality; or two or more severe adverse events for which a causal relationship with the investigational products cannot be ruled out.  2) The clinical trial will be discontinued if serious adverse events for which a causal relationship with the investigational products cannot be ruled out occur during the trial and fall under either of the following conditions: two or more subjects aged below 18 experience severe adverse event(s); or a subject aged below 18 experiences three or more adverse events. The trial can be continued, however, if the Independent Data Monitoring Committee concludes that there is clear justification and means to maintain safety of remaining subjects. |
| 1. Independent Data Monitoring Committee   In this clinical trial, an Independent Data Monitoring Committee shall be established for assessment of safety. The composition of committee members, matters to be assessed, and assessment criteria are to be arranged in accordance with procedures for the Independent Data Monitoring Committee separately prescribed. |
| 1. Target Study Sample Size   A sample size of 16 subjects is expected to be studied: 12 subjects for NPC-12T active substance arm, and 4 subjects for NPC-12T placebo arm.  [Reason for choice of sample size]  As this clinical trial will focus on a rare disease, and is exploratory, feasibility was the main aspect that was considered in setting the sample size. In terms of safety analysis, provided that clinically significant adverse events exist, the size is sufficient to detect adverse events of 15% incidence with 85% accuracy. In terms of efficacy analysis, when making a comparison of NPC-12T active substance arm concerning hearing test endpoints of periods without treatment and with treatment, a difference can be detected at 80% power (two-sided 5% significance level), if the number of evaluable subjects is 11 and Cohen’s d for endpoints is 0.85.  In estimation of correlation coefficients between evaluation indices, if data of two arms are integrated for evaluation, and the number of evaluable subjects having population correlation coefficient of 0.7 (i.e., medium to high degree of correlation) is 13, a difference can be detected at a minimum of 80% power (two-sided 5% significance level). |
| 1. Expected Duration of the Study   From March 2018 to November 2020 (approximately 2.5 year) (Registration: From March 2018 to December 2019 (approximately 22 months)) |

|  | Screening | Phase I (3 months) | | | | Phase II (6 months) | | | | | | | | Follow-up | Discontinuation |
| --- | --- | --- | --- | --- | --- | --- | --- | --- | --- | --- | --- | --- | --- | --- | --- |
| **Visits** | Visit 0 (V0) | V1 | V2 | V3 | V4 | V5^1,2^ | V6^2^ | V7^2^ | V8 | V9 | V10 | V11 | V12 | V13 |  |
| **Days/Week** | -2W | D1 | 2W | 4W | 8W | 12W | 14W | 16W | 20W | 24W | 28W | 32W | 36W | 40W |  |
| **Allowable visit windows (days)** | +/-7 | 0 | +/-3 | +/-3 | +/-7 | +/-3 | +/-3 | +/-3 | +/-7 | +/-7 | +/-7 | +/-7 | +/-7 | +/-7 |  |
| In-clinic visits | X | X | X | X | X | X | X | X | X | X | X | X | X | X | X |
| Informed consent | X |  |  |  |  |  |  |  |  |  |  |  |  |  |  |
| Eligibility judgment, random assignment, and prescription of investigational products | | | | | | |  |  |  |  |  |  |  |  |  |
| Eligibility judgment | X | X |  |  |  |  |  |  |  |  |  |  |  |  |  |
| Registration and assignment |  | X |  |  |  |  |  |  |  |  |  |  |  |  |  |
| Prescription of investigational products |  | X | X | X | X | X^1^ | X | X | X | X | X | X |  |  |  |
| Prior medication review |  | X | X | X | X | X | X | X | X | X | X | X | X |  | X |
| Tests to be carried out at subjects’ homes | | |  |  |  |  |  |  |  |  |  |  |  |  |  |
| Instructions on the portable test devices / ePRO | X |  |  |  |  |  |  |  |  |  |  |  |  |  |  |
| Test using the portable devices and ePRO input (at patients’ home) |  |  |  |  |  |  |  |  |  |  |  |  |  |  |  |
| Collection of data from portable devices and ePRO |  | X | X | X | X | X | X | X | X | X | X | X | X | X | X |
| Collection of the portable devices and ePRO |  | X^3^ |  |  |  |  |  |  |  |  |  |  |  | X | X |
| In-hospital tests and observation | |  |  |  |  |  |  |  |  |  |  |  |  |  |  |
| Medical interview on dizziness | X | X | X | X | X | X | X | X | X | X | X | X | X | X | X |
| Thoracic X-ray | X |  |  |  |  |  |  |  |  |  |  |  |  |  |  |
| Pure-tone audiometry | X | X |  | X | X | X |  | X | X | X | X | X | X | X | X |
| Auditory brainstem response | X |  |  |  | X |  |  |  |  |  |  |  | X |  | X |
| Posturography | X | X |  | X | X | X |  | X | X | X | X | X | X | X | X |
| Carolic and c-VEMP tests | X |  |  |  |  | X |  |  |  | X |  |  | X |  | X |
| Speech audiometry | X |  |  |  | X |  |  |  |  |  |  |  | X | X | X |
| Thyroid ultrasound | X |  |  |  |  | X |  |  |  |  |  |  | X |  | X |
| MRI (optional)^4^ |  |  |  |  |  | X^5^ |  |  |  |  |  |  | X^5^ |  | X^5^ |
| Cytology test (optional) |  |  |  |  |  | X |  |  |  |  |  |  | X |  | X |
| Blood collection for generation of iPS cells (optional)^6^ |  |  |  |  |  |  |  |  |  |  |  |  |  |  |  |
| Blood collection for measurement of drug concentration in blood |  |  |  |  |  | X | X | X | X | X | X | X | X |  | X |
| Clinical laboratory tests (blood sampling and urinalysis)^7^ | X | X |  | X |  | X | X | X | X |  | X |  | X | X | X |
| Physical findings (height and weight) | X | X |  |  |  | X |  |  |  |  |  |  | X |  | X |
| Blood pressure, pulse, and temperature | X | X | X | X | X | X | X | X | X | X | X | X | X |  | X |

^1^ The initial administration of investigational products in Phase II for respective subjects shall not be scheduled for the same day.

^2^ The interval between V5 and V6 and between V6 and V7 should be 10 days or more.

^3^ Applies when disqualified by the eligibility judgment.

^4^ An image taken at an external institution may be used if the date of imaging falls within the allowable window and is obtained with the consent of the patient.

^5^ The allowable visit windows are to be four weeks before V5, four weeks after V12, and four weeks after discontinuation. This examination may be performed on an alternative date as an exception.

^6^ If blood was collected in the past for generation of iPS cells, and consent for its use for the clinical trial has been obtained, blood collection need not be newly performed.

^7^ Hematocrit, serum triglyceride, viral tests, thyroid autoantibody tests, pregnancy test are performed at V0 only. Thyroid hormone tests are performed at V1, V5, V12, and discontinuation only.

# Background and Rationale

## Pendred syndrome/DFNB4

Pendred syndrome/DFNB 4 (PDS) is a disorder first described by Vaughan Pendred in 1896, the main symptoms of which include fluctuating and progressive hearing loss, persistent vertigo, and goiter (Pendred V, Lancet 1896; ii; 532). PDS is a rare genetic disorder caused by autosomal recessive disorder, and with an estimated 4,000 patients in Japan, it affects the second largest patient population among hereditary hearing disorders in the country. While the *SLC26A4* gene, which encodes pendrin, an anion exchanger, is known to be responsible for PDS (Everett LA, Nature Genetics. 1997; 17(4):411-22), the mechanism that leads to fluctuation and progressive cochlear disorder had long remained unknown. PDS patients with fluctuating hearing loss experience volatile changes in hearing acuity, and thus they suffer not only inconveniences in their daily life, but also fear of losing their ability to communicate orally with people around them following every acute exacerbation. Symptoms of PDS involve rotating vertigo that may last a few hours to a few days once it occurs, in addition to chronic dizziness, which are grave detriments to the patients’ quality of life.

No medication for hereditary hearing loss has pathophysiologically confirmed non-clinical POC anywhere in the world. For PDS, there is no effective treatment available at this time. The only effective interventions that exist are use of devices to augment hearing, such as hearing aids and cochlear implants. Although appropriate adjustment of a hearing aid requires consultation at a medical institution or with an audiologist, volatility in hearing and sporadic occurrence of fluctuations in a PDS patient impede the full performance of such adjustment. The hearing loss in PDS can progress as it fluctuates, and a PDS patient whose symptoms have progressed to severe hearing loss has an option to have a cochlear implant. Although the device provides a sense of sound, the sound quality is significantly deteriorated compared to what can be perceived with inner hair cells, as the number of electrodes used in a cochlear implant is limited to around twenty to date. With regard to vertigo, no medical intervention exists today, and thus patients have no option but to have bed rest and wait until the episode abates.

## Challenges in PDS Research and Background of Development of This Therapy (iPS Drug Discovery)

As described above, PDS is a rare and intractable disorder with no causal treatment, causing significant loss of patients’ QOL, and thus a new treatment has long been awaited. PDS is a hereditary disorder, and a knockout mouse for the *Slc26a4*, the responsible gene, develops severe hearing loss from severe malformation. A knock-in mouse for H723R mutations, the most commonly reported mutations in Japanese population with hearing impairment, however, does not develop deafness. This means that fluctuating and progressive hearing loss is not recapitulated in these mouse models. The lack of animal models is one of the impediments to understanding the pathology and developing treatments, and a reason that PDS treatments continue to depend on hearing devices.

We have described that this discrepancy is attributable to the species difference between primates and rodents, i.e., in primates, a family protein with similar functions and SLC26A4 co-express (Hosoya M et al, Neurosci. Res., 2016 Sep;110:1-10). This suggests that animal models generally used to demonstrate non-clinical POC in drug discovery research are essentially inapplicable to PDS due to species difference. Considering this fact, we have undertaken preclinical studies for iPSC-based drug discovery as part of research by Okano, a co-investigator, et al. at Keio Center for the Program for Intractable Diseases Research Utilizing Disease-specific iPS cells of AMED (Japanese Unexamined Patent Application Publication 2015-231365); identified pathophysiology of a neurodegenerative disorder that involves protein aggregation as a new pathophysiological mechanism of cochlear hearing loss; and performed drug screening with cell death suppression as metrics (Hosoya M et al., Cell Reports, 2017, 3;18(1):68-81). We have discovered in this research that patient-derived iPS cells of inner ear present intracellular aggregations of mutant protein and vulnerability to cellular stress, which shares similarities in physiopathology with Parkinson’s disease and Alzheimer’s disease. We further screened existing drugs based on this finding, and identified sirolimus, an mTOR inhibitor, as an inhibitor of cell death. The minimum effective concentration required for inhibition of cell death was 0.9 ng/mL, which was less than 1/10 of the approved dose of sirolimus preparation (PCT/JP2016/05086, WO2016/117431).

The natural history of inner ear disorder in PDS patients presents recurrent transient episodes and indolent progression. As no correlation is observed between inner ear malformation and hearing loss in PDS, hypofunction rather than malformation of the inner ear is assumed to be a therapeutic target for the disorder.

We constructed three different in vitro cell models that correspond to PDS pathophysiology, using the disease-specific cells generated from patient-derived iPS cells: (1) acute injury from exposure to high-concentration epoxomicin; (2) acute injury from exposure to low-concentration epoxomicin; and (3) chronic injury without exposure to any agents. Sirolimus 0.9 ng/mL significantly reduced cell death in all three models.

The results suggested low-dose sirolimus for PDS patients suffering from exacerbating and progressive symptoms aggravated by life stressors and head trauma may ameliorate vulnerability of the patient’s inner ear cells, and further suggested this may lead to attenuation of hearing loss and vertigo episodes and decelerated progression due to reduced cell death.

## Problems in Clinical Development for PDS Inner Ear Disorder

PDS is a rare disease, with an estimated patient population of around 4,000 in Japan. The lack of treatment, however, motivates few patients to consult doctors regularly and willingly. Existing reports are limited to those based on limited data collected from outpatients during office visits. Use of existing historical control data on natural course of changes in hearing in PDS therefore is assumed to be insufficient for evaluating the efficacy in clinical research. Newly collecting and accumulating such data would thus contribute to evaluation of efficacy in this trial, and will further serve as basic information in preparing protocols for research to be conducted.

This trial will collect a larger amount of information and more detailed data by measuring objective functions of the inner ear at patients’ homes, in addition to regular office visits, and explore what tests would enable the most precise evaluation. The trial is positioned as Phase I/IIa clinical research aimed at safety analysis and exploratory assessment of efficacy, including endpoints.

# Objective

To explore safety, tolerability, and efficacy of NPC-12T for Pendred syndrome/DFNB4 (PDS), for which treatment has yet to be established

# Study Plan

## Schedule of Activities

Table 1 shows the procedures and schedule of the trial.

Table 1 Procedures and Schedule

|  | Screening | Phase I (3 months) | | | | Phase II (6 months) | | | | | | | | Follow-up | Discontinuation |
| --- | --- | --- | --- | --- | --- | --- | --- | --- | --- | --- | --- | --- | --- | --- | --- |
| **Visits** | Visit 0 (V0) | V1 | V2 | V3 | V4 | V5^1,2^ | V6^2^ | V7^2^ | V8 | V9 | V10 | V11 | V12 | V13 |  |
| **Days/Week** | -2W | D1 | 2W | 4W | 8W | 12W | 14W | 16W | 20W | 24W | 28W | 32W | 36W | 40W |  |
| **Allowable visit windows (days)** | +/-7 | 0 | +/-3 | +/-3 | +/-7 | +/-3 | +/-3 | +/-3 | +/-7 | +/-7 | +/-7 | +/-7 | +/-7 | +/-7 |  |
| In-clinic visits | X | X | X | X | X | X | X | X | X | X | X | X | X | X | X |
| Informed consent | X |  |  |  |  |  |  |  |  |  |  |  |  |  |  |
| Eligibility judgment, random assignment, and prescription of investigational products | | | | | | |  |  |  |  |  |  |  |  |  |
| Eligibility judgment | X | X |  |  |  |  |  |  |  |  |  |  |  |  |  |
| Registration and assignment |  | X |  |  |  |  |  |  |  |  |  |  |  |  |  |
| Prescription of investigational products |  | X | X | X | X | X^1^ | X | X | X | X | X | X |  |  |  |
| Prior medication review |  | X | X | X | X | X | X | X | X | X | X | X | X |  | X |
| Tests to be carried out at subjects’ homes | | |  |  |  |  |  |  |  |  |  |  |  |  |  |
| Instructions on the portable test devices / ePRO | X |  |  |  |  |  |  |  |  |  |  |  |  |  |  |
| Test using the portable devices and ePRO input (at patients’ home) |  |  |  |  |  |  |  |  |  |  |  |  |  |  |  |
| Collection of data from portable devices and ePRO |  | X | X | X | X | X | X | X | X | X | X | X | X | X | X |
| Collection of the portable devices and ePRO |  | X^3^ |  |  |  |  |  |  |  |  |  |  |  | X | X |
| In-hospital tests and observation | |  |  |  |  |  |  |  |  |  |  |  |  |  |  |
| Medical interview on dizziness | X | X | X | X | X | X | X | X | X | X | X | X | X | X | X |
| Thoracic X-ray | X |  |  |  |  |  |  |  |  |  |  |  |  |  |  |
| Pure-tone audiometry | X | X |  | X | X | X |  | X | X | X | X | X | X | X | X |
| Auditory brainstem response | X |  |  |  | X |  |  |  |  |  |  |  | X |  | X |
| Posturography | X | X |  | X | X | X |  | X | X | X | X | X | X | X | X |
| Carolic and c-VEMP tests | X |  |  |  |  | X |  |  |  |  |  |  | X |  | X |
| Speech audiometry | X |  |  |  | X |  |  |  |  |  |  |  | X | X | X |
| Thyroid ultrasound | X |  |  |  |  | X |  |  |  | X |  |  | X |  | X |
| MRI (optional)^4^ |  |  |  |  |  | X^5^ |  |  |  |  |  |  | X^5^ |  | X^5^ |
| Cytology test (optional) |  |  |  |  |  | X |  |  |  |  |  |  | X |  | X |
| Blood collection for generation of iPS cells (optional)^6^ | X |  |  |  |  |  |  |  |  |  |  |  |  |  |  |
| Blood collection for measurement of drug concentration in blood |  |  |  |  |  | X | X | X | X | X | X | X | X |  | X |
| Clinical laboratory tests (blood sampling and urinalysis)^7^ | X | X |  | X |  | X | X | X | X |  | X |  | X | X | X |
| Physical findings (height and weight) | X | X |  |  |  | X |  |  |  |  |  |  | X |  | X |
| Blood pressure, pulse, and temperature | X | X | X | X | X | X | X | X | X | X | X | X | X |  | X |

^1^ The initial administration of investigational products in Phase II for respective subjects shall not be scheduled for the same day.

^2^ The interval between V5 and V6 and between V6 and V7 should be 10 days or more.

^3^ Applies when disqualified by the eligibility judgment.

^4^ An image taken at an external institution may be used if the date of imaging falls within the allowable window and is obtained with the consent of the patient.

^5^ The allowable visit windows are to be four weeks before V5, four weeks after V12, and four weeks after discontinuation. This examination may be performed on an alternative date as an exception.

^6^ If blood was collected in the past for generation of iPS cells, and consent for its use for the clinical trial has been obtained, blood collection need not be newly performed.

^7^ Hematocrit, serum triglyceride, viral tests, thyroid autoantibody tests, pregnancy test are performed at V0 only. Thyroid hormone tests are performed at V1, V5, V12, and discontinuation only.

### Expected Duration of the Study

March 2018 through November 2020 (approximately 2.5 years)

Subjects’ registration: March 2018 through December 2019 (approximately 22 months)

### Period for Administering the Investigational Products

NPC-12T tablets (active substance): 178 days maximum

NPC-12T tablets (placebo): 259 days maximum

### Clinical Trial Design

A phase I/IIa double-blind, parallel-group, single-institute trial initiated by a medical investigator

## Study Population

Outpatients with fluctuating hearing diagnosed as PDS in *SLC26A4* genetic testing are eligible to participate if all of the inclusion criteria described below are met, and if none of the exclusion criteria below are met.

[Inclusion Criteria]

1) Aged at least seven and below fifty years at the time of consent

2) Confirmed PDS-positive by genetic testing such as Sanger Sequencing

3) Report of subjective symptoms of fluctuating hearing disorder in a medical interview for case registration

4) Subjective symptoms of episodes of hearing loss and/or dizziness/vertigo within one year prior to the case registration

5) Had undergone inner-ear function testing at least three times, including standard pure-tone audiometry, performed with an interval of one day or longer, within six months prior to the case registration

6) Voluntary informed consent in writing obtainable from the patient or the patient’s legally acceptable representative

[Rationale]

1) To allow participation of patients of a wide range of ages, in consideration of patient demographics

2) through 5) To select patients who are considered to be appropriate for evaluation of investigational product efficacy

6) To ensure ethical conduct of the clinical trial

[Exclusion Criteria]

1) Prior use of molecular targeted drugs related to mTOR pathway, such as sirolimus, other mTOR inhibitors (e.g., everolimus) and tyrosine kinase (e.g., bevacizumab, sorafenib)

2) History of hypersensitivity to sirolimus, including sirolimus derivatives, and its additive agents

3) HBs antigen-positive, HBs antibody-positive, HBc antibody-positive, or active hepatitis C (HCV antibody-positive patients with inactive hepatitis and with normal-range values of liver function do not apply.)

HBs antigen-positive patients for which the cause is presumed to be due to vaccination against hepatitis B may participate in this clinical trial.

4) Severe blood abnormalities or hepatic dysfunction that demonstrate conditions shown below.

| Items | Value |
| --- | --- |
| AST or ALT | 100 IU/L or higher |
| Hematocrits | below 30% |
| Platelet count | below 80,000/mm^3^ |
| Absolute neutrophil count | below 1,000/mm^3^ |
| Total white blood cell count | below 3,000/mm^3^ |

5) Patients with poorly controlled dyslipidemia

Serum triglyceride of 500 mg/dL or higher or LDL cholesterol level of 190 mg/dL or higher despite being under treatment for dyslipidemia.

6) Severe renal impairment

Estimated glomerular filtration rate (eGFR) lower than 30 mL/min/1.73 m^2^

7) Immunodeficiency such as HIV or primary immunodeficiency

8) Inability to take investigational products in tablet form, or gastrointestinal dysfunction with risks of malabsorption of sirolimus

9) History of having surgery within eight weeks prior to the registration (any surgery that requires invasion of the body or suture with three or more stitches, including biopsy)

10) Female patients who are pregnant, potentially pregnant, or breastfeeding. Male or female patients who decline to consent to contraception during the trial

Contraceptive methods: Combination of condoms and spermicide; combination of condoms and diaphragms with spermicide; oral contraceptive; intrauterine contraceptive device; or other methods

11) Need to take medications that influence CYP3A4 activation after the administration of investigational products

12) Pulmonary interstitial opacity

13) Diagnosis by the attending physician during the screening phase or from previous observations from hearing tests that the hearing loss is fixed rather than fluctuating

14) Participation in other clinical research and/or trials within six months prior to the date of consent, with the exception of Analysis of Diseases in Otorhinolaryngology Using iPS Cells (approval No. 20140172)

15) Other conditions that the principal investigator or subinvestigator deems inappropriate

[Rationale]

1) through 7), 9) through 12), and 15) To ensure patients’ safety and to assess safety appropriately

8) The situation disturbs prediction of pharmacokinetics of the investigational products.

13) and 14) These may influence analysis of the investigational products.

# Endpoints

[Primary Endpoints]

Safety and tolerability:

1) Adverse events (nature, frequency and severity)

2) Side effects (nature, frequency and severity)

3) Clinical laboratory tests

4) Vital signs (blood pressure, pulse, and temperature)

[Secondary Endpoints]

Efficacy:

1) Frequency of hearing loss episodes (number of occurrences/year)

2) Highest measurement of auditory threshold shift at a hearing loss episode (dB)

3) Frequency of dizzy spells (number of occurrences/year)

4) Maximum amplitude (°/sec) and maximum frequency (cycle/sec) of nystagmus during a hearing loss episode

5) Percentage of cases in which increase in hearing threshold was observed during remission in the observation period (comparison of measurements at the end of Phase I and the end of Phase II)

6) Improvement in hearing threshold during remission

7) Shorter duration of hearing loss episodes

8) Alleviation of symptoms of dizziness/vertigo

Changes in total scores of the Dizziness Handicap Inventory from Phase I to Phase II

9) Percentage of cases in which equilibrium exacerbated during remission in the observation period (comparison of measurements at the end of Phase I and the end of Phase II)

[Exploratory Endpoints]

1) Reduction in endolymphatic hydrops (MRI findings)

2) Inhibition of goiter or thyroid enlargement

3) Cell biological changes in thyrocytes detected by cytology tests

4) Comparison of results of in vitro efficacy evaluation of inner ear cells derived from PDS-specific iPS cells and of clinical evaluation

5) Deviation from threshold value for frequency of abnormalities of auditory brain stem responses and for pure-tone audiometry

# Target Study Sample Size

A sample size of 16 subjects is expected to be studied: 12 subjects for the NPC-12T active substance arm, and 4 subjects for the NPC-12T placebo arm.

# Informed Consent Process

## Preparation of Written Information and Consent Form

The principal investigator will prepare a written information and consent form (informed consent form). The informed consent form will be provided as a set of documents consisting of written information and consent document, and will be revised as appropriate.

The informed consent form as prepared or revised will be submitted to the head of the institution for approval by the Institutional Review Board in advance of use.

## Elements to be Included in the Informed Consent Document

The written information shall include the following explanations, at minimum:

1) That the clinical trial involves research

2) The objectives of the clinical trial

3) The names, titles, and contact information of the principal investigator and subinvestigators

4) Clinical trial design, including experimental aspects and the inclusion and exclusion criteria

5) The expected clinical benefits, foreseeable risks, and inconveniences to the patients. If there is no intended clinical benefit to the patient, the patient shall be made aware of that information.

6) The alternative procedures or courses of treatment that may be available to the patient, and their important potential benefits and risks, if the patient is to become a subject

7) The expected duration of the patient's participation in the trial

8) That the patient’s participation in the trial is voluntary and that the patient may refuse to participate or may withdraw from the trial at any time, without penalty or loss of benefits to which the patient is otherwise entitled

9) That, by signing a written informed consent form, the patient will be granting access by the monitors, auditors, the Institutional Review Board, and regulatory authorities to the patient’s original medical records without violating the confidentiality of the patient

10) If the results of the trial are published, the patient’s identity will remain confidential

11) Information on whom to inquire or contact at the institution for further information regarding the trial and the rights of trial subjects, and in the event of trial-related injury

12) The compensation and/or treatments available to the patient in the event of trial-related injury

13) Type of the Institutional Review Board reviewing/deliberating the appropriateness of the clinical trial, etc., matters reviewed/deliberated by each Institutional Review Board, and other matters concerning the Institutional Review Board involved in the clinical trial

14) The approximate number of patients to be participating in the trial

15) That the patient will be informed in a timely manner if information becomes available that may be relevant to the patient’s willingness to continue participation in the trial

16) The foreseeable circumstances and/or reasons under which the patient’s participation in the trial may be terminated

17) The anticipated expenses, if any, to be borne by the patient while participating in the trial

18) The anticipated payment, if any, to made to the patient for participating in the trial

19) Matters that the patient is to comply with

## Procedure for Obtaining Informed Consent

1) Prior to the beginning of the trial, the principal investigator or subinvestigator will fully inform the patient or his/her legally acceptable representative of all pertinent aspects of the trial, presenting the informed consent form approved by the Institutional Review Board. Clinical research coordinators may assist in providing the information. The information will be provided in accordance with the informed consent form for this trial, using language as simple as practical to make sure that the patient or his/her legally acceptable representative understands it. Questions from the patient or his/her legally acceptable representative about the trial will be thoroughly answered. Upon confirmation that the patient or his/her legally acceptable representative well understands the information, a written voluntary consent to participation in the trial shall be obtained from the patient or his/her legally acceptable representative.

2) The informed consent form will be signed or have name seal affixed, and be personally dated by the principal investigator or subinvestigator who conducted the informed consent discussion, and by the patient or his/her legally acceptable representative. The clinical research coordinator who assisted in providing the information will also sign or affix their name seal, and personally date the form, if applicable.

3) The principal investigator or subinvestigator will deliver the signed/sealed and dated informed consent form to the patient or his/her legally acceptable representative prior to the patient’s participation in the trial. The original informed consent form will be stored appropriately in accordance with the policy of the institution.

4) Considering that the target population for the trial ranges from patients aged seven to forty nine years, an informed consent form concerning an underage patient, i.e., under twenty years of age, shall be accompanied by a written consent from the patient’s legally acceptable representative, who has been fully informed about the trial, along with reference to the informed consent form. The principal investigator or subinvestigator will also provide an explanation to the patient in plain language, and obtain written informed consent from the patient as far as practical.

## Revision of the Informed Consent Form

1) The principal investigator will determine in a timely manner whether the informed consent form requires any revision whenever important new information becomes available that may be relevant to the subjects’ consent.

2) If the principal investigator recognizes the need for revision, the informed consent form will be revised in a timely manner and receive renewed approval by the Institutional Review Board.

3) When a case to which 2) above, applies arises, the principal investigator or subinvestigator will inform the subjects already participating in the trial or their legally acceptable representatives orally of the situation without delay, ascertain whether the subjects are willing to continue their participation in the ongoing trial, and record the responses in subjects’ clinical records.

4) The principal investigator or subinvestigator shall provide an explanation to the subjects already participating in the trial or their legally acceptable representatives using the revised informed consent form approved by the Institutional Review Board, and obtain written voluntary consent from the subjects or their legally acceptable representatives in regard to subjects’ continued participation in the trial.

5) The principal investigator or subinvestigator, and the subject or his/her legally acceptable representative will sign/seal and personally date the consent form in the same manner as the initial consent. The clinical research coordinator who assisted in providing the information will also sign or affix their name seal, and personally date the form, where applicable.

6) The principal investigator or subinvestigator will deliver the signed/sealed and dated informed consent form to the subjects or their legally acceptable representatives. The original informed consent form will be stored appropriately in accordance with the policy of the institution.

# Investigational Products

## Brief Description of the Investigational Products

1) Test drug

(1) Product name: NPC-12T active substance tablets

(2) Ingredients: Sirolimus

(3) Dosage form: White, sugar-coated tablets containing sirolimus 1 mg each

(4) Storage: Stored in airtight containers, at ambient temperature, and away from light.

2) Comparator

(1) Product name: NPC-12T placebo tablets

(2) Dosage form: Tablets containing excipients identical to those of the active substance NPC-12T, but not containing sirolimus

(3) Storage: Stored in airtight containers, at ambient temperature, and away from light.

## Packaging and Labeling

The investigational product provider shall package and label the investigational products in the following manner for delivery to the storage manager(s) of the institution:

1) Packaging

<Phase I: Single-blind trial>

NPC-12T placebo tablets will be packaged in PTP (push-through-pack) sheets, with each sheet containing 10 tablets. A set of 10 PTP sheets will be wrapped in a transparent pillow package, which is then to be unit boxed and sealed.

<Phase II: Double-blind trial>

Test drug (silorimus): NPT-12T active substance tablets will be packaged in PTP sheets, with each sheet containing 10 tablets. A set of 10 PTP sheets will be wrapped in a transparent pillow package, which is to be unit boxed and sealed. Eight unit boxes will be packed in one inner carton.

Comparator (placebo): NPT-12T placebo tablets will be packaged in PTP sheets, with each sheet containing 10 tablets. A set of 10 sheets will be wrapped in a transparent pillow package, which is to be unit boxed and sealed. Eight unit boxes will be packed in one inner carton.

One set of investigational products dispensed to each subject will be put together in one master carton, which will consist of two unit boxes of Phase I placebo (200 tablets in total) and one inner carton of Phase II test drug or comparator (eight unit boxes containing 800 tablets in total).

2) Labeling

The labels on individual packets and unit boxes will explicitly indicate “for clinical trial use only,” and include at least the following information:

・That the products are solely for clinical trial use

・Code number of a treatment assignment

・The name, title, and address of the principal investigator

・Identification number

・Manufacturer’s serial number

・Storage

・Quantity

・Expiration

Unit boxes, inner cartons, and master cartons should be labeled as shown below.

The corner mark on the top right corner indicates the position where code number stickers should be placed.

(1) Unit boxes for Phase I

| For clinical trial use only Phase I NPC-12T tablets  Protocol Number: KCTR-D012  Manufacturer’s Serial Number: NP12T17X1  Storage: at room temperature  Quantity: 100 tablets (10 sheets)  Expiration: as prescribed in the policy on expiration of the investigational products  Principal investigator: Masato Fujioka, MD, PhD, Assistant Professor, Keio University School of Medicine  35 Shinano-cho, Shinjyuku-ku, Tokyo 160-8582  Note: The box and unused drugs are to be collected. Please do not discard. |
| --- |

(2) Unit boxes for Phase II (test drug and comparator)

| For clinical trial use only Phase II NPC-12T tablets  Protocol Number: KCTR-D012  Manufacturer’s Serial Number: NP12T17X1  Storage: at room temperature  Quantity: 100 tablets (10 sheets)  Expiration: as prescribed in the policy on expiration of the investigational products  Principal investigator: Masato Fujioka, MD, PhD, Assistant Professor, Keio University School of Medicine  35 Shinano-cho, Shinjyuku-ku, Tokyo 160-8582  Note: The box and unused drugs are to be collected. Please do not discard. |
| --- |

(3) Inner cartons for Phase II (containing eight unit boxes)

| For clinical trial use only Phase II NPC-12T tablets  Protocol Number: KCTR-D012  Manufacturer’s Serial Number: NP12T17X1  Storage: at room temperature  Quantity: 800 tablets (8 unit boxes)  Expiration: as prescribed in the policy on expiration of the investigational products  Principal investigator: Masato Fujioka, MD, PhD, Assistant Professor, Keio University School of Medicine  35 Shinano-cho, Shinjyuku-ku, Tokyo 160-8582  Note: The box and unused drugs are to be collected. Please do not discard. |
| --- |

(4) Master boxes for individual subjects (containing two unit boxes for Phase I and one inner carton for Phase II)

| For clinical trial use only NPC-12T tablets  Protocol Number: KCTR-D012  Manufacturer’s Serial Number: NP12T17X1  Storage: at room temperature  Quantity: 2 unit boxes for Phase I and 8 unit boxes for Phase II  Expiration: as prescribed in the policy on expiration of the investigational products  Principal investigator: Masato Fujioka, MD, PhD, Assistant Professor, Keio University School of Medicine  35 Shinano-cho, Shinjyuku-ku, Tokyo 160-8582  Note: The box and unused drugs are to be collected. Please do not discard. |
| --- |

## Handling of the Investigational Products

The principal investigator will procure investigational products after two weeks or later from the conclusion of the agreement with the investigational product provider regarding provision of the investigational products and the submission of the protocol. The investigational product storage manager will store and manage the investigational products in accordance with the Procedures for the Management of the Investigational Products developed by the principal investigator. After the end of the trial, the storage manager will dispose of any unused products appropriately and document the fact.

The investigational products shall not be used for any purposes other than this clinical trial.

# Procedures of the Trial

## Registration of Patients

The principal investigator or subinvestigator will obtain written informed consent from patients who are thought to satisfy the inclusion criteria, or their legally acceptable representatives, and register the patients in accordance with the following procedures:

| 1) Selection of patients | |
| --- | --- |
|  | The principal investigator or subinvestigator will carefully examine whether respective patients are eligible to participate in the clinical trial, taking account of patients’ health conditions, symptoms, age, gender, relationship with the principal investigator, and participation in any other clinical trials. |
| 2) Informed consent | |
|  | The principal investigator or subinvestigator will provide information pertinent to the clinical trial to patients who are thought to satisfy the inclusion criteria of the trial, and obtain written informed consent from the patients or their legally acceptable representatives. |
| 3) Assignment of subject identification code | |
|  | Subsequent to informed consent, required data will be entered into the electronic case report forms, and subject identification code numbers will be assigned. This will be recorded on the subject screening list. The code numbers will be used for identification of the patients throughout this trial. |
| 4) Screening | |
|  | Patients will go through screening two weeks (+/- seven days) before the initial intake of the investigational products. |
| 5) Confirmation of eligibility by the principal investigator or subinvestigator | |
|  | Prior to starting the first dose of the investigational products, the principal investigator or subinvestigator will judge whether patients satisfy the inclusion criteria, based on the assessment during the screening phase, using the eligibility check sheet. |
| 6) Registration of subjects and assignment of investigational products (V1) | |
|  | Patients whose eligibility to participate in the trial is confirmed in 5) will be registered and have investigational products assigned via the electronic case report form. Unique code numbers (pharmaceutical agent numbers) will be assigned to the registered subjects. This will be recorded in the subject screening log.  Patients whose eligibility is denied in 5) will be treated as screen failures. Screen failures will be recorded in the remarks column of the subject screening log. |

## Randomization and Blinding

1) Methods of assignment

The randomization manager will implement random assignment in accordance with the randomization procedure. When assigning NPC-12T placebo, the randomization manager will specify three types of dosage: reduction, increase, or no change in dosage. In specifying reduction or increase, specific visit(s) (V7 to V11) when the change in dosage is to be implemented will be indicated. The randomization manager will generate and store a randomization schedule, which is to be concealed. The randomization manager will generate prescription schedules containing information on assignment for respective subjects and specification for change in dosage of NPC-12T placebo tablets. The information for respective subjects will be sealed separately. The prescription schedules will be stored by an unblinded doctor. The randomization manager will store the randomization schedule in strict concealment until the time when the blind is permitted to be broken. When the unblinded doctor makes judgment on dosage change for a subject based on trough concentration measurements (V7 to V11), the doctor will unblind the prescription schedule for the subject in question and check the criteria for dosage change. The prescription schedule will be placed in concealment again immediately after the check. The unblinded doctor will keep a record every time the prescription schedule is unblinded or concealed. The randomization manager will generate two sets of emergency key codes when the assignment is made, which will be kept strictly in concealment by the randomization manager and the emergency contact center (the administrative office supporting clinical trial: Keio University Hospital Clinical and Translational Research Center) until the blind is authorized to be broken.

2) Indistinguishability

The randomization manager will verify the indistinguishability of medications and packages before random assignment and after the end of the trial.

3) Maintenance of blindness during the trial

With regard to the blood concentration of sirolimus, any person involved in the trial, with the exception of the unblinded doctor and unblinded clinical research coordinators (CRCs), will follow the procedures for prescription of investigational products to ensure blindness to the test results is maintained. The test results will be reported to the Independent Data Monitoring Committee and the principal investigator, however, after the blind is broken or if the principal investigator so directs in case of emergency. The unblinded CRCs will assist in the work of the unblinded doctor and make sure that the dosage change based on trough concentration measurements is appropriately instructed.

## Procedure for Concealing and Unblinding Randomized Code

The randomization manager will conceal and store the randomization schedule (randomized code) after the random assignment, and unblind it after the database is locked and the trial is completed.

Two sets of emergency key codes will be generated, which will be concealed by the randomization manager. The randomization manager and the emergency contact center (the administrative office supporting clinical trial: Keio University Hospital Clinical and Translational Research Center) will keep them in strict concealment until the appropriate time when the blind is permitted to be broken. When a serious adverse event or other situation demands urgent need for the principal investigator or subinvestigator to access information on assigned treatment to determine treatment of the subject, or when discovery of subject’s pregnancy requires revelation of assigned treatment, the principal investigator will determine whether the code should be partially unblinded. If the principal investigator deems it necessary to unblind the code assigned to the subject in question, the emergency contact center will be requested to allow partial unblinding of the randomization code (emergency key code). The partial unblinding of the emergency key code will be conducted in accordance with the procedure manual for unblinding.

The emergency contact center will communicate the unblinded information to the principal investigator. If partial unblinding is performed, the principal investigator will document justification for unblinding and the extent to which the information is made available.

# Administration of Investigational Products

Figure 1 Dosing Protocol

Phase I (single-blind): NPC-12T placebo tablets for 3 months (12 weeks)

Phase II (double-blind): NPC-12T active substance tablets or NPC-12T placebo tablets for 6 months (24 weeks)

[Dosage at the outset of the trial]

Subjects weighing less than 40 kg will take one 1-mg tablet orally once every other day.

Subjects weighing 40 kg or more will take one 1-mg tablet orally once per day.

The investigational products are to be taken before sleep.

The bodyweight measured at V1 will be referred to in determining dosage throughout the trial.

<Rationale for the dosing time>

The package insert of Rapamune 1 mg tablets was referred to when setting the dosing time. The insert cites reports that state administration of the tablet after a high-fat meal increased the blood concentration of sirolimus. To reduce effects of high-fat meals, the dosing time of the investigational products is set to be before sleep.

[Prescription of the investigational products]

In Phase I (V1, V2, V3, and V4) and Phase II (V5 and V6), the principal investigator or subinvestigator will prescribe investigational products in accordance with the dosage at the start of the trial described above.

In Phase II (V7, V8, V9, V10, and V11), the unblinded doctor will instruct the principal investigator and subinvestigators on the prescription of the investigational product based on the criteria for dosage change. The principal investigator or subinvestigator will make a judgment on whether dosage reduction is required or dosage increase is permissible, taking account of subjects’ safety, including risks of adverse events. If the conditions are deemed appropriate, the principal investigator or subinvestigator will prescribe the investigational products as instructed by the unblinded doctor. The unblinded doctor will provide instructions to the principal investigator or subinvestigator in accordance with the trough concentration measurements and the prescription schedule generated by the randomization manager. The trough concentration measurements and the prescription schedule are not to be disclosed to any person except the unblinded CRCs until the end of the trial, and will be kept securely locked in a cabinet accessible only to the unblinded doctor.

[Criteria for dosage change]

From Phase II, the dosage will be determined taking account of the trough concentration measurements from the last visit (V6, V7, V8, V9, and V10) and occurrence of adverse events.

The unblinded doctor will provide instructions to the principal investigator or subinvestigator on prescription of the investigational products based on the trough concentration measurements at visits.

The principal investigator or subinvestigator will make a judgment on whether dosage reduction is required or dosage increase is permissible before prescribing, with due consideration for the subjects’ safety, such as risks of adverse events.

In this trial, the therapeutic range of the trough concentration following the administration of NPC-12T active substance is set to be 2.5 to 7.5 ng/mL. The trough concentration at the last visit below 2.5 ng/mL will be considered to be suitable for dosage increase, and that exceeding 7.5 ng/mL for dosage reduction.

Dosage increase for NPC-12T active substance will be permitted only if the principal investigator or subinvestigator deems it appropriate from the viewpoint of subject safety. The unblinded doctor will instruct the principal investigator or subinvestigator to prescribe the investigational products not exceeding two 1-mg tablets per day (i.e., 2 mg/day), administered orally.

The reduced dosage of NPC-12T active substance should be no less than one 1-mg tablet every three days, administered orally. If further reduction is required, administration of the investigational product to the subject will be discontinued.

As dosage adjustment according to trough concentration is unsuitable for the NPC-12T placebo arm, one of increase, reduction, or no change in dosage is specified for the placebo arm at the time of assignment of the investigational products. At the same time, the randomization manager will designate the visits for dosage change for the placebo arm.

Dosage increase for NPC-12T placebo will be permitted only if the principal investigator or subinvestigator deems it appropriate from the viewpoint of subject safety. When dosage increase is specified in the prescription schedule, the unblinded doctor will instruct the principal investigator or subinvestigator to prescribe a dosage level at one tier higher.

If the prescription schedule specifies reduction, the unblinded doctor will instruct the principal investigator or subinvestigator regarding dosage reduction for NPC-12T placebo. The dosage level will be lowered by one tier at a time. The reduced dosage must be no lower than one 1-mg tablet every three days, administered orally. If further reduction is required, administration of the investigational products will be discontinued.

If the principal investigator or subinvestigator determines further reduction is required, administration of the investigational products will be discontinued.

The unblinded doctor will provide instructions to the principal investigator or subinvestigator on prescription in accordance with the specified dosage change in the prescription schedule. The decision by the principal investigator or subinvestigator on permission for dosage increase and reduction shall take precedence over the specification in the prescription schedule, i.e., if the principal investigator or subinvestigator specifies reduction, and the prescription schedule states increase, reduction should be adopted.

[Acceptable change in dosage]

The amounts of investigational products to be administered can be increased or decreased to one of the following dosages:

A. Two 1-mg tablets, once per day, administered orally

B. One 1-mg tablet, once per day, administered orally

C. One 1-mg tablet, once every other day, administered orally

D. One 1-mg tablet, once every three days, administered orally

[Dosage reduction or increase]

When dosage is to be increased, the dosage level should be raised by one tier at a time: Subjects weighing 40 kg or over will follow the change from B to A. Subjects weighing below 40 kg will follow the change from C to B to A.

When dosage is to be reduced, the dosage level should be lowered by one tier at a time, from A to B to C to D. If the current level for a subject is B, for example, C will be applied in the next reduction. If further reduction below D is required, administration of the investigational products to the subject will be discontinued.

[Suspension or reduction of administration of investigational products due to adverse events]

・Unacceptable adverse events or exacerbation considering the safety of the subject

・Unacceptable abnormal observations in clinical tests considering the safety of the subject

If a situation described above arises, dosing will be suspended until the relevant symptoms abate. The dosage will then be reduced by one tier at a time from A to B to C to D. If the sirolimus dosage is 1 mg/day every three days, administered orally, which corresponds to D, the dosing will be discontinued, and follow-up observations will be performed one month after that, before discontinuation of the patient’s participation in the trial, in principle.

The initial administration of investigational products in Phase II for respective subjects shall not be performed on the same day.

# Prohibited Concomitant Medications and Therapies

1) In principle, subjects will be prohibited from taking any of the following medications unless the principal investigator or subinvestigator allows the concomitant use in response to acute exacerbation:

ATP solution (e.g., Adetphos^Ⓡ^), Vitamin B12 solution (e.g., Methycobal^Ⓡ^), Betahistine (e.g., Merislon), and medications that improve microcirculation (e.g., Kallikrein).

2) Subjects will be prohibited from concomitant use of medications described below.

Sirolimus is metabolized by a drug-metabolizing enzyme CYP3A4, and is a substrate for P-glycoprotein. Sirolimus itself is an inhibitor of CYP3A4. Subjects are thus prohibited from taking any of the medications and foods listed below throughout their participation in this clinical trial, i.e., from the initial administration of investigational products (V1) to the end of the administration (V12).

| Influencing factors | Medications and food | Rationale |
| --- | --- | --- |
| 1) Inhibition of mTOR | everolimus; temsirolimus | The products have the same mechanism of action as the investigational products, which would disturb efficacy analysis. |
| 2) Influence on the assessment of the disease studied | aminoglycoside class of antibiotics; platinum-based drugs | Ototoxicity of the products could exacerbate inner ear disorder.  (Topical administration is allowed.) |
| 3) Live vaccines | live vaccines (e.g., measles, mumps, rubella, oral polio, BCG) | The immunosuppresive properties of sirolimus may cause proliferation of microorganisms in live vaccines leading to enhanced virulence.  (The package insert of Rapamune states these as contraindications for concomitant use.) |
| 4) Induction of CYP3A4 | anticonvulsants (e.g., carbamazepine, phenobarbital, phenytoin); rifampicin*; rifabutin; foods containing St. John’s Wort (*Hypericum perforatum*) | The products induce CYP3A4 and may lower blood concentrations of the investigational products.  (*Rifampicin induces P-glycoprotein and thus may stimulate removal of the investigational products from cells.) |
| 5) Inhibition of CYP3A4 | ciclosporin**; diltiazem**, triazole antifungal (e.g., fluconazole, ketoconazole**, itraconazole, voriconazole); HIV protease inhibitors (e.g., indinavir, ritonavir, telaprevir, saquinavir); macrolide class of antibiotics (e.g., erythromycin, clarithromycin); calcium channel blocker (e.g., nicardipine, verapamil); cimetidine; metoclopramide; bromocriptine; danazol; grapefruit juice | The products significantly inhibit CYP3A4, and may raise blood concentrations of the investigational products.  (Topical administration is allowed.)  (**These products inhibit P-glycoprotein and thus may hinder removal of the investigational products from cells.) |
| 6) Inhibition of CYP3A4 by ritonavir | ombitasvir hydrate; paritaprevir hydrate; ritonavir | Inhibition of CYP3A4 by ritonavir may raise AUC and C_max_ of the investigational products. |
| 7) Mechanism unidentified | micafungin sodium | The product may increase AUC of the investigational products. |
| 8) Other effects | angiotensin-converting-enzyme inhibitor | For a patient taking medications related to angioedema, the product may increase risks of onset of angioedema (e.g., swelling of face, lips, tongue, or throat). (Mechanism unidentified) |

# Oversight of Subjects

The principal investigator, subinvestigator, or clinical research coordinators will instruct the subjects on the following matters:

1) To undergo medical examinations and tests on designated dates. If the subject is unable to visit the institution on the date specified, the subject should contact the principal investigator, subinvestigator, or clinical research coordinator and follow their instructions.

2) To report to the principal investigator, subinvestigator, or clinical research coordinator regarding the use of all medications prescribed by doctors not involved in this trial and those purchased from pharmacists

3) Not to change their normal lifestyle, such as exercise or dietary habits, as far as possible

4) To contact the principal investigator, subinvestigator, or clinical research coordinator immediately in case any indisposition is felt

5) To adopt highly effective contraception during participation in the trial

# Tests, Observations, and Assessment

## Schedule for Tests, Observations, and Assessment

The items to be covered in tests and observations, and the schedule of the trial are shown in Table 1.

Tests and observations, and entry of the results into the electronic case report forms, can be performed by the clinical research coordinator under the oversight of the principal investigator or subinvestigator.

## Subjects’ Baseline Information

The principal investigator or subinvestigator will collect the baseline information listed below from each subject during the period between informed consent and before administration of the investigational products in V1 (D1), and record the information on the subject’s electronic case report form. For the purpose of this protocol, an illness that has not subsided before administration of the investigational products is referred to as a concomitant illness, and one that has subsided as a past medical history. The results of SLC26A4 genetic testing will not be recorded on the electronic case report form.

・Date of birth

・Gender

・Height and weight

・History of head trauma

・Concomitant illness

・Prior treatment (cisplatin and gentamicin)

・Concomitant medications

・SLC26A4 Genetic testing results

## Concomitant Medications and Therapies

The principal investigator or subinvestigator will examine concomitant medications and therapies from the screening phase (V0) to V13, and record the results on the electronic case report forms.

## Medication Adherence

The principal investigator or subinvestigator will inquire subjects about medication adherence from V1 to V12 and record the results on the respective electronic case report forms.

## Clinical Questionnaire

The principal investigator or subinvestigator will ask subjects to complete the questionnaire specified below (ePRO) every day, in principle, from the screening phase (V0) to V13, and record the results on the electronic case report forms.

・Questionnaire on daily conditions (Annex 2)

Medication adherence, hearing loss episodes, vertigo episodes, tinnitus, ear fullness, concomitant medications, handicaps caused by dizziness/vertigo (action, feeling, and daily life)

The principal investigator or subinvestigator will ask subjects to complete the Dizziness Handicap Inventory at every visit from the screening phase (V0) to V13 and at discontinuation, and record the results on the electronic case report forms.

・Dizziness Handicap Inventory: DHI-J (Annex 3)

## Tests Using Portable Testing Devices

The principal investigator or subinvestigator will provide subjects training on the portable devices and make adjustments to the devices during the screening phase (V0). Subjects will be asked to perform tests using the portable devices listed below from V0 to V13 every night, in principle. The test results will be collected at every visit from V1 to V13 and at discontinuation.

・Portable audiometer

Audiometer AA-58 (RION Co., Ltd.) (Annex 4)

For standard pure-tone audiometry (threshold of four different frequencies for left and right ears) and recording data in ePRO.

・Wireless Frenzel scope

AirMicro Frenzel Wireless Scope (Scalar Corporation) (Annex 5)

For tests such as measurement of rotational eye movement, and direction and amplitude of nystagmus, the results of which shall be transmitted to an Android terminal for storage.

The tests will be performed regularly in the evening and as appropriate when an episode occurs, as far as possible.

・JINS MEME

JINS MEME ES_R (JIN Co., Ltd.) (Annex 6)

For tests with open and closed eyes, each lasting around 30 seconds, the results of which shall be transmitted to an Android terminal for storage.

## In-clinic Testing and Observations

### Vital Signs

The principal investigator or subinvestigator will measure the vital signs listed below during the screening phase (V0), Phase I (V1 to V4), Phase II (V5 to V12), and in case of discontinuation, and record the results on the electronic case report forms.

・Blood pressure (systolic and diastolic blood pressure)

・Pulse

・Temperature

### Physical Findings

The principal investigator or subinvestigator will measure the physical findings listed below during the screening phase (V0), Phase I (V1), Phase II (V5 and V12) and in case of discontinuation, and record the results on the electronic case report forms.

・Height

・Weight

### Laboratory Tests

The principal investigator or subinvestigator will perform the following laboratory tests during the screening phase (V0), Phase I (V1 and V3), Phase II (V5, V6, V7, V8, and V12), follow-up observations (V13), and at discontinuation, and record the results on the electronic case report forms.

[Laboratory test items]

| Tests | Variables |
| --- | --- |
| Hematological tests | red blood cell count, hemoglobin, reticulocyte count, white blood cell count, platelet count, lymphocyte count, neutrophil count, and hematocrit (V0 only) |
| Blood biochemical tests | creatinine, ALP (Changes in the level of ALP will be analyzed as a blood marker), total bilirubin, AST, LDH, ALT, BUN, albumin, CRP, total cholesterol, LDL-cholesterol, uric acid, Na, K, Ca, P, Cl, fasting blood glucose, and serum triglyceride (V0 only) |
| Thyroid hormone tests | FT3, FT4, and TSH (all of these are performed at V1, V5, V12, and discontinuation only) |
| Urinalysis | pH, sugar, protein, and occult blood in urine |
| Viral test | HBs antigen, HBs antibody, HBc antibody, HCV antibody, and HIV antibody (all of these will be performed at V0 only) |
| Thyroid autoantibody tests | TgAb, TPOAb, and TRAb (all of these will be performed at V0 only) |
| Pregnancy test | hCG urine (V0 only) |

### Thoracic Radiography

The principal investigator or subinvestigator will perform thoracic radiography during the screening phase (V0) to examine incidence of pulmonary interstitial opacity, and record the results on the electronic case report forms.

### Pure-tone Audiometry

The principal investigator or subinvestigator will perform pure-tone audiometry during the screening phase (V0), Phase I (V1, V3, and V4), Phase II (V5, V7, V8, V9, V10, V11, and V12), the follow-up phase (V13), and at discontinuation, and record the results on the electronic case report forms.

### Auditory Brainstem Response

The principal investigator or subinvestigator will perform an auditory brainstem response test during the screening phase (V0), Phase I (V4), and Phase II (V12), and record the results on the electronic case report forms.

### Posturography

The principal investigator or subinvestigator will perform posturography during the screening phase (V0), Phase I (V1, V3, and V4), Phase II (V5, V7, V8, V9, V10, V11, and V12), the follow-up phase (V13), and at discontinuation, and record the results on the electronic case report forms.

### Caloric Test

The principal investigator or subinvestigator will perform a caloric test during the screening phase (V0), Phase II (V5, V9, and V12), and at discontinuation, and record the results on the electronic case report forms.

### c-VEMP Test

The principal investigator or subinvestigator will perform a c-VEMP test during the screening phase (V0), Phase II (V5, V9, and V12), and at discontinuation, and record the results on the electronic case report forms.

### Speech Audiometry

The principal investigator or subinvestigator will perform speech audiometry during the screening phase (V0), Phase I, (V4), Phase II (V12), and at discontinuation, and record the results on the electronic case report forms.

## Blood Concentration of Sirolimus

The principal investigator or subinvestigator will collect blood for analysis of blood concentration of sirolimus during Phase II (V5, V6, V7, V8, V9, V10, V11, and V12). Analysis of the blood will be entrusted to an external contractor.

## Exploratory Tests and Observations

### Collection of Blood for Generation of iPS Cells and in vitro Efficacy Testing of Sirolimus (Optional)

The principal investigator or subinvestigator will collect blood to be used for generation of iPS cells between V1 and V12. Generation of iPS cells and in vitro efficacy testing using PDS-specific iPS cells will be performed at Keio University School of Medicine.

Prior to performing this test, the subjects or their legally acceptable representatives will be provided information for consent separately from the informed consent for participation in the trial. Blood sample collection and in vitro efficacy testing will be carried out only for those who consent to the test.

### Thyroid Ultrasound

The principal investigator or subinvestigator will perform thyroid ultrasound during the screening phase (V0), Phase II (V5 and V12), and at discontinuation.

### MRI (optional)

The principal investigator or subinvestigator will perform an MRI scan of the inner ear during Phase II (V5 and V12) and at discontinuation for subjects aged 16 or older with consent to the test, four hours after intravenous injection of contrast media. The allowable visit windows are to be within four weeks before V5, four weeks after V12, and four weeks after discontinuation.

### Cytology Test (optional)

The principal investigator or subinvestigator will perform a cytology test of thyroid for an exhaustive gene expression analysis in Phase II (V5 and V12) and at discontinuation for subjects with consent to blood collection for cytology test. This study will be performed only for subjects with consent based on prior explanation to the subjects or their legally acceptable representatives. As soon as the specifics of the exhaustive gene manifestation mapping are established, an “informed consent form concerning exhaustive gene manifestation mapping using thyroid cytology test” will be prepared and the information will be provided to obtain consent.

## Adverse Events

### Definition of Adverse Events

An adverse event in this trial refers to any untoward medical occurrence in a subject from initial administration of investigational products (V1) to follow-up observations (V13). The event may not necessarily have to have a causal relationship with administration of investigational products in this trial. An adverse event can therefore be any unfavorable and unintended sign (including an abnormal laboratory finding, for example), symptom, or disease temporally associated with the use of a medicinal product, whether considered related to the medicinal product or not.

Exacerbation of the primary disease, including episodes, will not be regarded as an adverse event, with the exception of cases in which causal relationship with the investigational products cannot be ruled out.

### Assessment of Adverse Events

The items listed below will be examined and recorded on the electronic case report forms with regard to adverse events that occur from the initial administration of investigational products (V1) to follow-up observations (V13).

If a diagnosis is settled based on signs or symptoms, the diagnosis rather than the signs or symptoms should be recorded. With regard to vital signs and clinical test measurements, changes from conditions before administration of investigational products should be assessed to determine whether the changes are clinically significant. An abnormal change that is considered clinically significant is to be counted as an adverse event. An abnormal change that is relevant to symptoms and signs already recognized as an adverse event, shall not be required to be newly reported as a separate adverse event.

・Name of the adverse event

・Date of occurrence

・Severity

・Seriousness

・Actions taken (administration of investigational products or other actions)

・Outcome and date of outcome confirmation

・Causal relationship with the investigational products

### Definition of Serious Adverse Events

Seriousness of adverse events is categorized as follows:

1) Non-serious: cases other than 2)

2) Serious: events for which any of the items from a) to g), below, apply.

a) Death

b) Cases that might result in death

c) Any case that requires hospitalization for treatment or prolongs the duration of hospitalization

d) Disability

e) Cases that might result in disability

f) Other medically serious condition for which seriousness is equivalent to a) to e), above

g) Any congenital diseases or abnormalities in the next generation

### Causality Assessment

Causality is categorized into “related” or “unrelated,” which are defined below. An adverse event that is considered to be “related” to the investigational products is regarded as a side effect.

[Criteria for judging causality]

| Causality | Description |
| --- | --- |
| Related | There is a reasonable possibility that the adverse event is related to the investigational products. |
| Unrelated | Conditions other than the above. |

### Severity of Adverse Event

Adverse events and severity of each adverse event will be assessed in accordance with the grades below.

[Grading scale for adverse events]

| Grade 1 | Mild; asymptomatic or mild symptoms; clinical or diagnostic observations only; or intervention not indicated. |
| --- | --- |
| Grade 2 | Moderate; minimal, local or noninvasive intervention indicated; or limiting age-appropriate instrumental activities of daily living (ADL)*. |
| Grade 3 | Severe or medically significant but not immediately life-threatening; hospitalization or prolongation of hospitalization indicated; disabling; or limiting self care ADL**. |
| Grade 4 | Life-threatening consequences; urgent intervention indicated. |
| Grade 5 | Death related to adverse event. |

*Instrumental ADL refers to preparing meals, shopping for groceries or clothes, using the telephone, managing money, etc.

**Self-care ADL refers to bathing, dressing and undressing, feeding self, using the toilet, taking medications, and not bedridden.

### Reporting of Serious Adverse Events

If a serious adverse event is observed, the principal investigator or subinvestigator shall take necessary measures such as treatment for the subject, produce a report concerning the serious adverse event, and report immediately to the head of the institution and the investigational product provider. In addition, the principal investigator shall consider whether the case should be reported to the Minister of Health, Welfare, and Labor. If it is determined that it should be, it shall be reported to the Japan Agency for Medical Research and Development.

The head of the institution shall report to the Institutional Review Board upon receipt of a report by the principal investigator on a serious adverse event, and provide directions to the principal investigator regarding continuation of the trial based on the view of the Institutional Review Board.

### Follow-up of Adverse Events

The principal investigator or subinvesigator will follow up all adverse events that occur during the clinical trial until the event has resolved or recovered to the state before the event, in principle. If there is a reasonable ground that justifies termination of the follow-up before completion, the principal investigator or subinvestigator will record the justification on the electronic case report forms on termination.

If an adverse event develops into a serious adverse event during the follow-up, the principal investigator shall report the situation promptly to the head of the institution and the investigational product provider, and act in accordance with Section 14.10.6, “Reporting of Serious Adverse Events.”

### Provision of Additional Information

1) If the principal investigator becomes aware of any information regarding serious and unpredictable side effects as stipulated in Article 80-2 (6) of the Law on Securing Quality, Efficacy and Safety of Products Including Pharmaceuticals and Medical Devices (Pharmaceuticals and Medical Devices Law) or Article 273 of the Enforcement Regulations of the Pharmaceuticals and Medical Devices Law, the principal investigator shall report the fact to the head of the institution and the regulatory authorities within the specified period.

The head of the institution will make a decision on whether continuation of the clinical trial is appropriate, after consultation with the Institutional Review Board. The principal investigator will provide updated information to the subjects in accordance with Section 8.4, “Revision of the Informed Consent Form.”

2) In addition to 1) above, the head of the institution will submit to the Institutional Review Board any report from the principal investigator concerning revision of the investigator’s brochure and the protocol, revision of the informed consent form, matters related to quality, efficacy, and safety of the investigational products, important information for appropriate operation of the clinical trial, and revision of other documents on matters to be discussed by the Institutional Review Board.

### Pregnancy during the Trial

If a subject or her partner is found to be pregnant or potentially pregnant, the principal investigator or subinvestigator will follow-up until delivery and assess the condition of the mother and child after the end of pregnancy, including delivery, as far as practical. If the subject herself is pregnant, administration of the investigational products to the subject will be immediately terminated. Unblinding of the emergency key code will be requested as appropriate.

## Discontinuation Criteria

### Participant Withdrawal

The principal investigator or subinvestigator will withdraw a subject from the trial if he/she meets any of the criteria listed below. On withdrawal of the clinical trial, the principal investigator or subinvestigator will immediately secure the safety of the subject and take appropriate measures. When a subject is withdrawn from the clinical trial, reasons for the withdrawal and occurrence of adverse events will be identified and recorded in the source document and on the electronic case report form. Tests that are scheduled at discontinuation will be performed as far as practical.

1) Subject’s request for withdrawal from the trial

2) Subject no longer trackable

3) Discovery of obvious ineligibility for participation in the trial

4) Decision of the principal investigator or subinvestigator that the subject’s participation in the trial would be difficult due to an adverse event that occurs after the initiation of the trial

5) Pregnancy of the subject

6) Death of the subject

7) Need for dosage reduction below the lowest standard of one 1-mg tablet every three days, administered orally

8) Other conditions that the principal investigator or subinvestigator deems appropriate for withdrawal

### Discontinuation of the Clinical Trial

The principal investigator and subinvestigators will take measures described below during the trial. If the trial itself is to be discontinued, the principal investigator and subinvestigators should maintain the safety of the subjects and take other appropriate measures. Reasons for discontinuation and any adverse events should be identified and recorded in the original documents and on the electronic case report forms. Tests and analyses that are scheduled at discontinuation will be performed as far as practical.

1) The Independent Data Monitoring Committee will be convened to review appropriateness of continuance of the trial, if adverse events of the following types occur: one or more serious adverse events regardless of causality; or two or more severe adverse events for which a causal relationship with the investigational products cannot be ruled out.

2) The clinical trial will be discontinued if serious adverse events for which a causal relationship with the investigational products cannot be ruled out occur during the trial and fall under either of the following conditions: two or more subjects aged below 18 experience severe adverse event(s); or a subject aged below 18 experiences three or more adverse events. The trial can be continued, however, if the Independent Data Monitoring Committee concludes that there is clear justification and means to maintain safety of remaining subjects.

# Independent Data Monitoring Committee

## Establishment of the Independent Data Monitoring Committee

In this clinical trial, an Independent Data Monitoring Committee shall be established for assessment of safety. The composition of committee members, matters to be assessed, and assessment criteria are to be arranged in accordance with procedures for the Independent Data Monitoring Committee separately prescribed.

## Purpose of the Independent Data Monitoring Committee

The Independent Data Monitoring Committee will be organized by the principal investigator to review appropriateness of continuance of the clinical trial to ensure reasonable safety of subjects.

## Assembly of the Independent Data Monitoring Committee

[Calling committee meetings]

If any of the significant matters listed below occurs, the principal investigator will promptly request the chair of the Independent Data Monitoring Committee to call a committee meeting to review appropriateness of continuance of the trial and other related matters.

1) Discovery of new significant information on safety concerning the investigational products of the trial

2) Serious adverse events in the trial, regardless of causality

3) Recognition of new, significant information on safety derived from similar products, research reports, or other sources

4) Other matters that the principal investigator deems essential

## Meeting Format and Discussion Materials for the Independent Data Monitoring Committee

The meetings of the Independent Data Monitoring Committee will be convened with the participation of all committee members, including participation via the Web.

The sponsor investigator will submit the materials below to the Independent Data Monitoring Committee for discussion.

[Materials for a primary discussion]

Any of the following materials as appropriate:

1) Reports concerning serious adverse events (Form 12)

2) Reports concerning discontinuation of production, recall, or disposal of the investigational products abroad

3) Summary of the latest report on the safety of the investigational products

4) Investigational products, side effects, and case reports in Japan

5) Individual Report Common Line Listing; and a list of unexpected and serious adverse drug reactions

6) Significant updates on safety information of similar products from research publications or other materials

7) Other information of which the sponsor investigator requested assessment

8) Reports on severe adverse events. This may be replaced by copies of the electronic case report forms.

[Materials for a secondary discussion]

Any of the following materials as appropriate, in addition to the materials for the primary discussion:

1) Copies of electronic case report form pages containing adverse events, vital signs, and clinical test results after the completion of SDV of cases registered

2) Data on blood concentrations of sirolimus

3) A duplicate copy of a report for partial unblinding of the emergency key codes

4) Other information as committee members deem required

## Criteria of Judgment by the Independent Data Monitoring Committee

[Judgment Criteria]

1) Continuation of the clinical trial will not be authorized if adverse events for which a causal relationship with the investigational products cannot be excluded occur during the trial and fall under either of the following conditions: two or more subjects aged below 18 experience severe adverse event(s); or a subject aged below 18 experiences three or more adverse events. The trial can be continued, however, if the Independent Data Monitoring Committee concludes that there is clear justification and means to maintain safety of remaining subjects.

2) Continuation of the clinical trial will not be authorized if new significant information on safety derived from similar products, research reports, or other sources is considered to be applicable to the trial.

# Compliance with, Deviation from, Changes in, or Amendment to the Protocol

## Compliance with the Protocol

The principal investigator and the subinvestigator shall abide by this protocol in implementing the clinical trial.

## Protocol Deviations or Changes

The principal investigator and subinvestigators shall not deviate from or make any changes to the protocol without prior written approval by the Institutional Review Board after consultation, unless there is an imminent danger to a subject that has to be avoided or any other situation medically justifiable, or a change related solely to administrative matters is involved.

If the principal investigator deviates from the protocol on grounds of avoidance of imminent danger to a subject or other medically justifiable situation, the principal investigator will describe the justification and relevant matters in a report concerning deviation from the protocol for avoiding an imminent danger. The report will be submitted to the head of the institution, and a copy of the report will be kept by the principal investigator. The principal investigator and subinvestigators will record all deviations from the protocol, regardless of the cause. The subject or his/her legally acceptable representative may refuse to participate or may withdraw from the clinical trial at any time. The subject’s refusal or withdrawal will not result in penalty or loss of benefits to which the subject is otherwise entitled.

# Statistical Analyses

## Populations for Analyses

### Analysis Sets

[Safety]

The safety analysis set will include a set of subjects to whom the investigational products had been administered at least once.

[Efficacy]

1) Full Analysis Set (FAS)

The full analysis set (FAS) will be based on the intention-to-treat (ITT) principle, and will include all patients in this trial other than those who fall under any of the following conditions:

・Violation of eligibility criteria (patients who do not meet main registration criteria for this trial)

・Patients in whom the investigational products had never been administered

・Non-existence of baseline nor treatment data

・Withdrawal of consent during the trial and refusal to allow use of all data

2) Per Protocol Set (PPS)

The Per Protocol Set will include a set of subjects who meet the eligibility criteria for the efficacy analysis based on the standards for subject analysis sets developed before database locking. Eligibility criteria for data to be measured chronologically shall be specified for individual points in time for measurement.

The main analysis set for efficacy in this trial is to be the FAS. For main efficacy endpoints, statistical analysis of the PPS will also be performed. Comparison of the results of two distinctive analysis sets will be used to evaluate the robustness of main analysis results of the trial.

### Data Handling

Standards for analysis sets will be established before database lock.

## Demographic Variables and Baseline

Summary statistics of the subjects’ basic data (e.g., gender, age, and weight) and baseline will be calculated for each dosage arm.

## Statistical Analysis of Efficacy

Assessment of efficacy in this trial is regarded as a secondary objective. With regard to continuous endpoints, summary statistics of changes or percentage changes based on actual measurements and from baseline at each point in time will be calculated for each dosage arm. A one-sample *t*-test will be used for comparison between actual measurements and baseline values at each point in time for each dosage arm. The two-sided 95% confidence interval for average change for each dosage arm will then be estimated. Using a two-sample *t*-test, the change or percent change at each point in time between the two arms will be compared. The two-sided 95% confidence interval for average difference between the two arms will be estimated. If the data is found to be non-normal, use of nonparametric methods and statistical methods based on probability distribution other than normal distribution will be considered. With regard to binary endpoints, a contingency table will be compiled for each point in time, and Fisher’s exact test will be applied in making comparison of the distribution in the two dosage arms. The Clopper-Pearson method will be applied to estimate the two-sided 95% confidence interval for each dosage arm. The difference in the two-sided 95% confidence interval will be estimated using the normal approximation.

### Efficacy Endpoints

[Secondary Endpoints]

1) Frequency of hearing loss episodes (number of occurrences per year)

2) Highest measurement of auditory threshold shift at a hearing loss episode (dB)

Definition of hearing loss episodes: Hearing loss episodes with subjective symptoms and episodes detected as audiometric data will be counted as hearing loss episodes in this trial. Concerning audiometric data, audiometric testing of four different frequencies for right and left ears will be performed, and the average of measurements obtained at five recent time points will be defined as the baseline. A change of 10 dB or more observed in the testing period including the screening phase will be identified as an episode. The period from the beginning of the episode until the condition returns to the baseline will be counted as one episode. Episodes observed in left and right ears or in different frequencies concurrently will be considered as separate episodes.

3) Frequency of dizzy spells (number of occurrences per year)

Dizzy spells in which the subject is conscious are to be counted.

4) Maximum amplitude (°/sec) and maximum frequency (cycle/sec) of nystagmus in a hearing loss episode

5) Percentage of cases in which increase in hearing threshold was observed during remission in the observation period (comparison of measurements at the end of Phase I and the end of Phase II)

6) Improvement in threshold during remission

7) Shorter duration of hearing loss episodes

8) Alleviation of symptoms of dizziness/vertigo

Changes in total scores of the Dizziness Handicap Inventory from Phase I to Phase II

9) Percentage of cases in which equilibrium exacerbated during remission in the observation period (comparison of measurements at the end of Phase I and the end of Phase II)

[Exploratory Endpoints]

1) Reduction in endolymphatic hydrops (MRI findings)

2) Inhibition of goiter or thyroid enlargement

3) Cell biological changes in thyrocytes detected by cytology tests

4) Comparison of results of in vitro efficacy evaluation of inner ear cells derived from PDS-specific iPS cells and of clinical evaluation

5) Deviation from threshold value for frequency of abnormalities of auditory brain stem responses and for pure-tone audiometry

## Statistical Analysis of Safety

### Adverse Events

The number of occurrences and types of adverse events and of side effects will be sorted by clinical symptoms and by abnormal change of clinical test results. A two-sided 95% confidence interval of the incidence rate by respective dosing arms will be calculated using the Clopper-Pearson method.

### Clinical Test Results and Vital Signs

With regard to continuous endpoints, actual measurements, summary statistics of amounts or rates of change, and two-sided 95% confidence intervals will be calculated for each dosing arm. With regard to discrete variables, cross tables of baselines and individual time points will be created for each dosing arm.

## Target Study Sample Size

A sample size of 16 subjects is expected to be studied: 12 subjects for NPC-12T active substance arm, and 4 subjects for NPC-12T placebo arm.

[Reason for choice of sample size]

As this clinical trial will focus on a rare disease, and is exploratory, feasibility was the main aspect that was considered in setting the sample size. In terms of safety analysis, provided that clinically significant adverse events exist, the size is sufficient to detect adverse events of 15% incidence with 85% accuracy. In terms of efficacy analysis, when making a comparison of NPC-12T arms concerning hearing test endpoints of periods without treatment and with treatment, a difference can be detected at 80% power (two-sided 5% significance level), if the number of evaluable subjects is 11 and Cohen’s d for endpoints is 0.85.

In estimation of correlation coefficient between evaluation indices, if data of two arms are integrated for evaluation, and the number of evaluable subjects having population correlation coefficient of 0.7 (i.e., medium to high degree of correlation) is 13, then a difference can be detected at a minimum of 80% power (two-sided 5% significance level).

## Significance Level and Multiplicity

The level of significance used for all analyses in this trial is to be two-sided 5%. The confidence coefficient of confidence interval is to be two-sided 95%. As the efficacy analysis in this trial is for a secondary purpose, multiplicity of different endpoints and time points will not be adjusted.

## Interim Analyses

No interim analysis will be performed.

## Deviations from the Original Statistical Analysis Plan

If an analysis is performed using a method different from the original analysis plan stated in the protocol, all changes should be reported in the clinical study report.

## Statistical Analysis Plan

A statistical analysis plan containing detailed scheduling of statistical analyses, revision, and data handling will be prepared before the unblinding. The unblinding will be performed after all cases are locked.

# Electronic Case Report Forms

## Entry into and Reporting via the Electronic Case Report Forms

The case reports will be produced in an electronic format, for which the principal investigator and clinical research coordinators will input data in the Electronic Data Capture (EDC) system. The clinical research coordinators shall be allowed to enter only transcriptions of data recorded in the source documents. The data entry manual for the electronic case report forms shall be followed with regard to methods of entry, revision, and correction. The subinvestigators and clinical research coordinators must be registered on a list of recognized subinvestigators and clinical research coordinators in order to enter information into an electronic case report form.

## Principal Investigator’s Confirmation of the Electronic Case Report From

Entries made by a subinvestigator or a clinical research coordinator will be checked by the principal investigator after every entry or before database locking.

The principal investigator will examine the data input in the EDC system and add an electronic signature upon acknowledgement of accuracy and completeness of all data, including audit trail and query management.

If any discrepancy between the data recorded in the EDC system and the source data is found, the principal investigator will document the reasons and keep the record in an appropriate manner.

If data managers of the CRO or monitors find any discrepancy or doubt about data recorded through data cleaning or SDV, regardless of whether the principal investigator had checked it, they will issue a query and request recheck, addition, revision or correction as appropriate.

## Database Locking and Unlocking

The data managers will lock the database in accordance with the data management procedure manual after the completion of data cleaning of the electronic case report form by the CRO and the electronic signature by the principal investigator acknowledging all entries in the form.

No addition, revision, or correction will be allowed after the database locking. If a need for correction arises after locking, the data managers will unlock the database in accordance with the data management procedure manual. The unlocking of the database will allow procedures described in the preceding section.

## Entry in the Questionnaire and Testing and Report Using a Portable Device

Subjects or their legally acceptable representatives will complete the questionnaire concerning the daily life of the subject (Section 14.5) every day, in principle, via the ePRO system, and bring the ePRO to the specified visits. The principal investigator or subinvestigator will collect the ePRO at every visit specified, check data entries, and question the subjects or their legally acceptable representatives regarding the entries as appropriate. If a shortcoming is found, the subjects or their legally acceptable representatives will be asked to revise or correct the entry. The principal investigator or subinvestigator will record the ePRO results on the electronic case report forms at every visit.

Subjects or their legally acceptable representatives will perform tests every day, in principle, using portable testing devices (Section 14.6), enter the results in the electronic devices, and bring all of the electronic devices on the specified visit dates. The portable audiometer measurements will be recorded as an ePRO report, and the wireless Frenzel scope and JINS MEME results will be recorded in an Android device. The principal investigator or subinvestigator will check the entries in the devices at every visit.

## Materials Recorded Directly on the Case Report Forms and Materials Regarded as Source Data/Documents

The information recorded on the electronic case report forms for the items listed below will be regarded as the source data.

1) Purpose of using concomitant medications and of performing concomitant therapies

2) Adverse events, including their seriousness, severity, outcomes, date of outcomes, causal relationship with the investigational products, and evidence of the causal relationship

3) Date of and reasons for discontinuation; adverse events that led to discontinuation; progress after the discontinuation; and follow-up observations

4) Comments by the principal investigator and subinvestigators

## Direct Access to Source Data/Documents

The principal investigator and the head of the institution will accept monitoring, audits, and investigations by the Institutional Review Board and regulatory authorities, and guarantee direct access to all source documents associated with the clinical trial.

# Quality Management and Assurance

The monitors will make sure that human rights, safety, and well-being of the subjects are preserved and that this clinical trial is compliant with GCP, the protocol, and standard operational procedures, in accordance with the procedure manuals set by the principal investigator. The monitors will monitor accuracy and completeness of data reported by the principal investigator and subinvestigators, with direct access to documents related to the trial, including the source data/documents.

The principal investigator will delegate auditing to auditors who are independent of ordinary monitoring and quality assurance of the trial, to assure that the trial complies with all regulatory requirements, GCP, the protocol, and standard operational procedures.

# Ethical Considerations

The trial shall be conducted in compliance with standards stipulated in Article 14-3 and Article 80-2 of the Pharmaceuticals and Medical Devices Law, the Ministerial Ordinance on Good Clinical Practice (GCP) for Drugs, and the protocol, respecting the ethical principles founded in the Declaration of Helsinki.

## Institutional Review Board

The Institutional Review Board will review matters concerning implementation and continuance of the clinical trial from ethical, scientific, and medical perspectives, based on the investigator’s brochure, the protocol, and informed consent forms.

## Patient Confidentiality

Patients will be identified by their subject identifier code on their registration forms and on case report forms. All persons involved in this trial shall preserve the confidentiality of the patients when accessing source data/documents associated with the implementation of the trial, in publication in medical journals, and upon submission of documents to regulatory authorities.

# Record Keeping

1) Records to be retained at the institution

The person responsible for storage of records designated by the head of the institution will retain required documents and records until date (1) or (2) below, whichever comes later. If the principal investigator demands a longer retention period, the institution will discuss the appropriateness of the retention period and methods with the principal investigator.

If the investigational product provider concludes that data concerning test results such as clinical study results will not be attached to a written application, the investigational product provider will notify the head of the institution about that fact and provide supporting reasons.

(1) The day on which marketing approval for the investigational product is obtained; the date of partial change of approved matters, if it relates to additional indications; or if a notification is issued that the development of the investigational products is to be discontinued or that the trial results are not to be attached to a written application, the day three years from the date of receipt of the notification.

(2) The day fifteen years from the date of discontinuation or completion of the clinical trial.

If the investigational product provider obtains marketing approval for the investigational products or chooses to terminate the development of the product before obtaining approval, the provider will report the fact in writing to the head of the institution.

2) Records to be retained by the principal investigator

The principal investigator will retain required documents and records until date (1) or (2) below, whichever comes later, in accordance with the procedure for standard operation of record keeping and documents management.

(1) The day five years from obtaining marketing approval for the investigational product; the day three years after the date of authorizing discontinuation, if the development of the investigational product was chosen to be discontinued; or the day of completion of a reexamination, if reexamination is required under the Pharmaceuticals and Medical Devices Law and the period for completing the reexamination exceeds five years.

(2) The day three years from the date of discontinuation or completion of the clinical trial.

If the investigational product provider obtains marketing approval for the investigational product or chooses to terminate the development of the product before obtaining marketing approval, the provider will report this fact in writing to the head of the institution.

# Financial assistance

Patients will be offered a certain amount of financial support to cover the auxiliary costs associated with participation in this trial. The expenses for medical treatment in this trial will be covered by health insurance in line with the system of the Medical Expenses Combined with Treatment Outside Insurance Coverage. The investigational products used in this trial will be provided free of charge by the investigational product provider.

The expenses for operating this clinical trial will be funded by the Japan Agency for Medical Research and Development.

It has been confirmed that these present no conflicts of interest, as concluded by the Conflict of Interest Management Committee of Keio University.

# Compensation for Adverse Health Effects

If a health hazard arises in a patient as a result of participation in this trial, the institution shall perform necessary and appropriate measures such as provision of medical treatment. If the principal investigator considers that the health hazard arose from the proper use of the investigational products and there is a causal relationship with the investigational products, the principal investigator will bear the compensation. Medical expenses and medical allowances will not be included in the compensation. Compensation may not be offered if it is judged that the health hazard is due to intentional or serious negligence of the patient.

# Publication Policy

Information, including data, obtained in this clinical trial may not be disclosed in whole or part regardless of the medium used, without prior consent from the sponsor investigator and the investigational product provider.

# References

Pendred V. Deaf mutism and goitre. Lancet 1896;II:532.

Everett LA, Glaser B, Beck J, Idol JR, Buchs A, Heyman M, et al. Pendred Ssyndrome is caused by mutations in a putative sulphate transporter gene (PDS). Nature Genetics 1997; 17:411-22.

Hosoya M, Fujioka M, Kobayashi R, Okano H, Ogawa K. Overlapping expression of anion exchangers in the cochlea of a non-human primate suggests functional compensation. Neurosci. Res., 2016 Sep; 110:1-10

Hosoya M, Fujioka M, Sone T, Okamoto S, Akamatsu W, Ukai H, Ueda HR, Ogawa K, Matsunaga T, Okano H. Cochlear Cell Modeling Using Disease-Specific iPSCs Unveils a Degenerative Phenotype and Suggests Treatments for Congenital Progressive Hearing Loss. Cell Reports, 2017, 3; 18(1):68-81

Jacobson GP, Newman CW. The development of the dizziness handicap inventory. Arch Otolaryngol Head Neck Surg, 1990; 116:424-7

Masuda M, Goto F, Fujii M, Kunihiro T. Investigation of the Reliability and Validity of Dizziness Handicap Inventory (DHI) Translated into Japanese. Equilibrium Res, 2004; 63 (6):555-63

# Organization of the Trial Site

See Annex 1.

# List of Annex

Annex 1 Institution Implementing the Clinical Trial

Annex 2 Questionnaire on Daily Condition

Annex 3 Dizziness Handicap Inventory

Annex 4 Instruction Manual for Audiometer AA-58 (RION Co, Ltd.)

Annex 5 Instruction Manual for AirMicro Frenzel Wireless Scope (Scalar Corporation)

Annex 6 Instruction Manual for JINS MEME ES_R (JIN Co., Ltd.)

Annex 7 Pregnancy Report Form
